# Immunization with a self-assembling nanoparticle vaccine displaying EBV gH/gL protects humanized mice against lethal viral challenge

**DOI:** 10.1016/j.xcrm.2022.100658

**Published:** 2022-06-14

**Authors:** Harman Malhi, Leah J. Homad, Yu-Hsin Wan, Bibhav Poudel, Brooke Fiala, Andrew J. Borst, Jing Yang Wang, Carl Walkey, Jason Price, Abigail Wall, Suruchi Singh, Zoe Moodie, Lauren Carter, Simran Handa, Colin E. Correnti, Barry L. Stoddard, David Veesler, Marie Pancera, James Olson, Neil P. King, Andrew T. McGuire

**Affiliations:** 1 Vaccine and Infectious Disease Division, Fred Hutchinson Cancer Research Center, Seattle WA 98109, USA; 2 Department of Biochemistry, University of Washington, Seattle, WA 98195, USA; 3 Institute for Protein Design, University of Washington, Seattle, WA 98195, USA; 4 Clinical Research Division, Fred Hutchinson Cancer Research Center Seattle, WA 98109, USA; 5 Division of Basic Sciences, Fred Hutchinson Cancer Research Center, Seattle, WA 98109, USA; 6 Howard Hughes Medical Institute, Chevy Chase, MD 20815, USA; 7 Department of Global Health, University of Washington, Seattle, WA 98195, USA; 8 Department of Laboratory Medicine and Pathology, University of Washington, Seattle WA 98115, USA

**Keywords:** vaccines, antibodies, Epstein-Barr virus, nanoparticles, immunity, gH/gL

## Abstract

Epstein-Barr virus (EBV) is a cancer-associated pathogen responsible for 165,000 deaths annually. EBV is also the etiological agent of infectious mononucleosis and is linked to multiple sclerosis and rheumatoid arthritis. Thus, an EBV vaccine would have a significant global health impact. EBV is orally transmitted and has tropism for epithelial and B cells. Therefore, a vaccine would need to prevent infection of both in the oral cavity. Passive transfer of monoclonal antibodies against the gH/gL glycoprotein complex prevent experimental EBV infection in humanized mice and rhesus macaques, suggesting that gH/gL is an attractive vaccine candidate. Here, we evaluate the immunogenicity of several gH/gL nanoparticle vaccines. All display superior immunogenicity relative to monomeric gH/gL. A nanoparticle displaying 60 copies of gH/gL elicits antibodies that protect against lethal EBV challenge in humanized mice, whereas antibodies elicited by monomeric gH/gL do not. These data motivate further development of gH/gL nanoparticle vaccines for EBV.

## Highlights

• Design of several self-assembling nanoparticles displaying EBV gH/gL
• Demonstrate multivalent display of gH/gL results in superior immunogenicity in mice
• Nanoparticle-elicited antibodies protect humanized mice against lethal EBV challenge

## Graphical abstract

**Figure:**
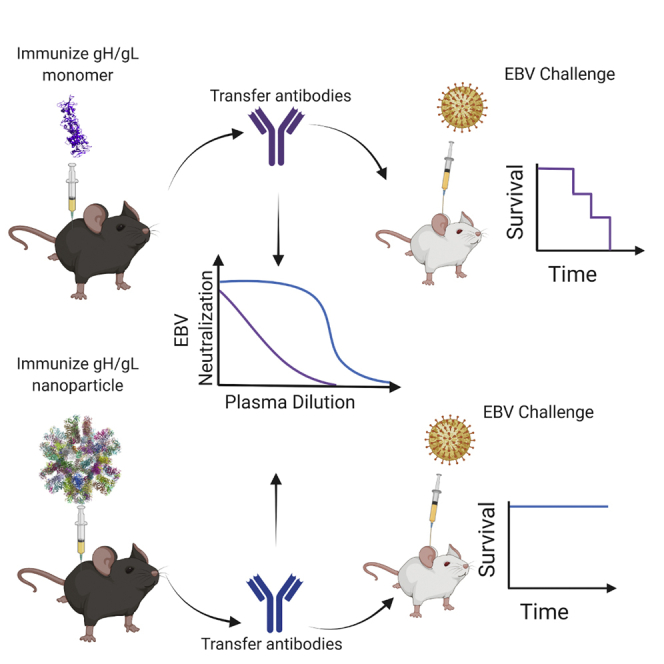

## Teaser

EBV is a cancer-associated pathogen for which there is no vaccine. Here, Malhi et al. develop nanoparticles displaying the viral gH/gL glycoprotein complex that elicit potent neutralizing antibody responses capable of protecting against lethal EBV challenge in humanized mice. These results support the use of gH/gL nanoparticles for EBV vaccine development.

## Introduction

Epstein-Barr virus (EBV) is one of the most common human viruses. It is a herpesvirus with tropism for both B cells and epithelial cells and is associated with several malignancies of these two cell types including Hodgkin lymphoma, Burkitt lymphoma, diffuse large B cell lymphoma, post-transplant lymphoproliferative disease, nasopharyngeal carcinoma, and gastric carcinoma.^1^, ^2^, ^3^, ^4^ It is estimated that EBV is responsible for ∼265,000 new cases of cancer and ∼164,000 cancer deaths globally per year.^1^^,^^5^, ^6^, ^7^ EBV is also the causative agent of infectious mononucleosis (IM) and is linked to multiple sclerosis and rheumatoid arthritis.^8^, ^9^, ^10^, ^11^, ^12^, ^13^, ^14^ Thus, a vaccine that prevents EBV infection and/or associated pathologies would have a significant global health impact.^1^^,^^6^^,^^15^

EBV is orally transmitted, and both B cells and epithelial cells are present in the oropharynx. Thus, an effective vaccine would likely need to prevent or severely limit infection in both cell types.^2^^,^^16^ The dual tropism of EBV infection is accomplished through the orchestrated function of multiple glycoproteins.^17^ gH, gL, and gB constitute the core fusion machinery and are essential for viral entry irrespective of cell type. gB is a transmembrane fusion protein that promotes the merger of the viral and host membranes.^18^ gB activity depends on the heterodimeric gH/gL complex, which regulates fusion and is essential for infection.^19^, ^20^, ^21^, ^22^ Epithelial cell infection is initiated by the binding of the viral BMRF-2 protein to β1 integrins on the cell surface.^23^ Following attachment, binding of gH/gL to one or more cell-surface receptors is thought to induce a conformational change that triggers gB activation. αvβ6, and αvβ8 integrins, neuropilin 1, non-muscle myosin heavy chain IIA, and the ephrin A2 receptor have all been implicated as gH/gL receptors.^24^, ^25^, ^26^, ^27^, ^28^, ^29^

Viral attachment to B cells is mediated by gp350, which binds to complement receptors (CRs) 1 and 2.^30^, ^31^, ^32^ The triggering of gB during B cell entry depends on the tripartite complex of gH/gL and the viral glycoprotein gp42. Binding of gp42 to the B chain of human leukocyte antigen class II leads to activation of gB through the gH/gL/gp42 complex.^33^, ^34^, ^35^

Neutralizing antibodies are the correlate of protection for most effective vaccines.^36^^,^^37^ It is therefore likely that they will be an important component of an immune response elicited by an EBV vaccine. Serum from naturally infected individuals can neutralize EBV infection of B cells and epithelial cells,^23^^,^^38^, ^39^, ^40^ and all the viral proteins involved in viral entry are targeted by neutralizing antibodies.^23^^,^^41^, ^42^, ^43^ To date, most EBV subunit vaccine efforts have focused on gp350. gp350 is capable of adsorbing most of the serum antibodies that neutralize EBV infection of B cells.^41^^,^^43^

Mechanistically, neutralizing anti-gp350 monoclonal antibodies (mAbs) block the gp350-CR1/CR2 interaction.^31^^,^^44^, ^45^, ^46^, ^47^ However, antibodies against gp350 are ineffective at inhibiting EBV infection of CR^-^ epithelial cells and can enhance infection of this cell type.^23^^,^^48^^,^^49^ Passive transfer of a neutralizing anti-gp350 mAb protected one of three macaques against high-dose experimental infection with rhesus lymphocryptovirus, the EBV ortholog that infects macaques^50^ indicating that gp350 antibodies could be protective *in vivo.* A phase II trial of a gp350 vaccine failed to protect against EBV despite decreasing the incidence of symptomatic IM by 78%.^51^ In light of these results, it has been suggested that a gp350 vaccine could be improved upon with the inclusion of additional viral proteins.^52^ Alternatively, it is possible that a vaccine targeting non-gp350 viral proteins could be more efficacious.

gH/gL is a promising antigen for vaccine development. Anti-gH/gL antibodies account for most serum antibodies that neutralize EBV infection of epithelial cells, but only a small fraction of antibodies that neutralize infection of B cells.^43^ Only a handful of anti-gH/gL mAbs have been identified, all of which neutralize EBV infection of epithelial cells with comparable potency, but most have weak or no neutralizing activity against EBV infection of B cells.^48^^,^^53^, ^54^, ^55^, ^56^, ^57^, ^58^ We previously described the isolation and characterization AMMO1, an anti-gH/gL mAb that potently neutralizes EBV infection of epithelial cells and B cells *in vitro* by binding to a discontinuous epitope on gH/gL.^55^ The 769B10 mAb also neutralizes EBV infection of both cell types and binds to an epitope that overlaps with AMMO1, confirming that this is a critical site of vulnerability on EBV.^43^ Passive transfer of AMMO1 severely limits viral infection following high-dose experimental EBV challenge in humanized mice and protects rhesus macaques against oral challenge with RhLCV if present at adequate levels at the time of challenge.^58^^,^^59^ These studies provide proof of concept that anti-gH/gL antibodies can protect against EBV infection and indicate that a gH/gL-based vaccine capable of eliciting AMMO1-like antibodies could prevent oral transmission of the virus.

Here, we generated several protein subunit vaccines where gH/gL is scaffolded onto self-assembling multimerization domains to produce nanoparticles with well-defined geometries and valency. Relative to monomeric gH/gL, immunization with the gH/gL nanoparticles elicited higher binding titers and neutralizing titers after one or two immunizations in mice. Competitive binding and depletion of plasma antibodies with an epitope-specific gH/gL probe suggested that only a small fraction of vaccine-elicited antibodies targeted the AMMO1 epitope. Consistent with this, depletion of plasma antibodies with an epitope-specific gH/gL knockout reduced plasma neutralizing activity to undetectable levels. Passive transfer of immunoglobulin G (IgG) purified from animals immunized with a computationally designed nanoparticle displaying 60 copies of gH/gL protected against high-dose lethal challenge in a humanized mouse model, while IgG purified from animals immunized with monomeric gH/gL did not. Collectively, these results demonstrate that gH/gL is an attractive vaccine antigen but that multivalent display of gH/gL is required to elicit neutralizing antibodies of sufficient titer to protect against EBV infection.

## Results

### Generation and characterization of multimeric gH/gL vaccine constructs

**Figure 1:**
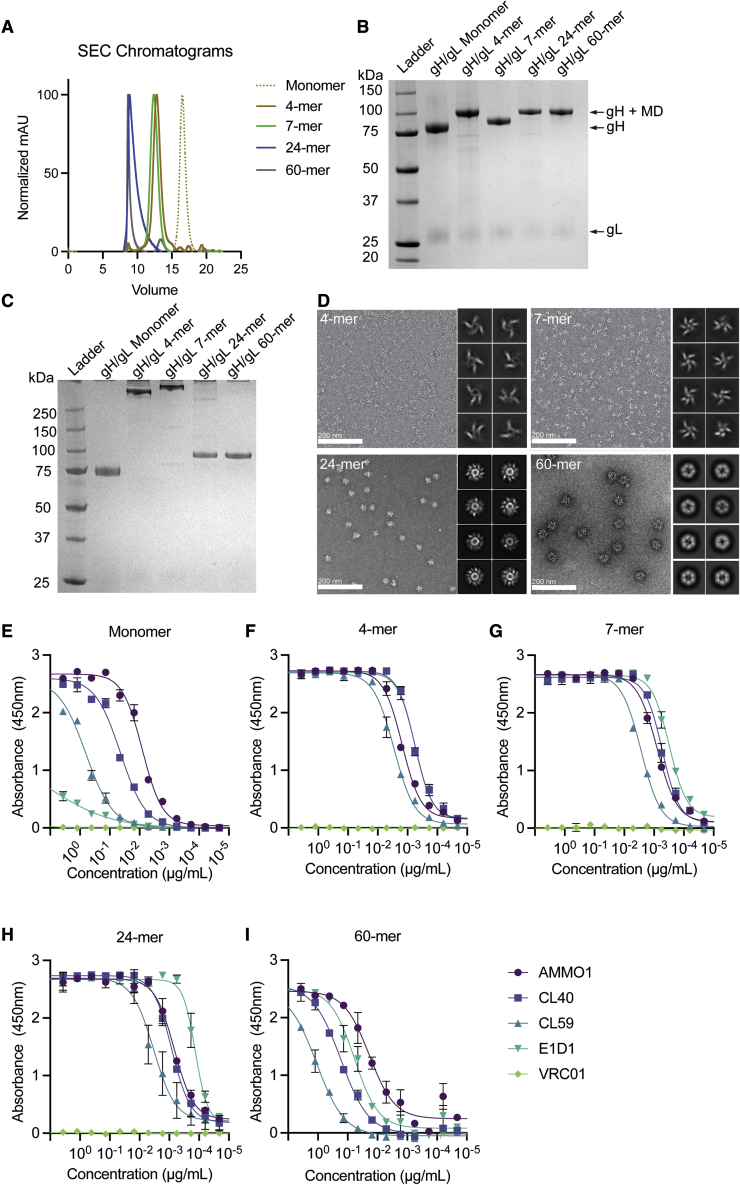
Biochemical and biophysical characterization of multimeric gH/gL nanoparticles (A) Monomeric gH/gL and multimeric gH/gL nanoparticles were analyzed by size-exclusion chromatography (SEC) on a Superose 6 column as indicated. (B) Reducing SDS-PAGE analysis of 1 μg of monomeric gH/gL or multimeric gH/gL nanoparticles. Bands corresponding to gL, gH, and gH fused to 4-, 7-, 24-, or 60-mer multimerization domains (MDs) are indicated with arrows. (C) Non-reducing SDS-PAGE analysis of 1 μg of the proteins in (B). (D) Negative-stain electron microscopy was performed on 4-, 7-, 24-, or 60-mer gH/gL nanoparticles as indicated. The eight most frequent 2D class averages for each particle are shown in the inlay. Scale bars represent 200 nm. (E–I) Binding of the anti-gH/gL mAbs E1D1, CL40, CL59, and AMMO1 to monomeric gH/gL (E) or multimeric gH/gL nanoparticles (F–I) were measured by ELISA as indicated. Each data point represents the mean, and error bars represent the standard deviation of two technical replicates. The anti-HIV-1 Env mAb VRC01 was used as a control for non-specific binding. See also Tables S1 and S2.

Cui et al. and Bu et al. have shown that immunization with multimeric gH/gL elicits higher serum neutralizing titers against infection of B cells and epithelial cells than immunization with monomeric gH/gL.^43^^,^^60^ However, these studies focused on a single multimerization platform when generating gH/gL constructs, either *Helicobacter pylori* ferritin, a 24-mer, or a T4 fibritin foldon domain, a trimer. Here, we sought to develop several self-assembling multimeric gH/gL constructs with differing valencies, sizes, and geometries to evaluate how they differ in their ability to elicit neutralizing antibodies in mice. We generated various expression constructs where different multimerization domains were genetically fused to the C terminus of the gH ectodomain. These included (1) a computationally designed circular tandem repeat protein (cTRP) that forms a planar toroid displaying four copies of gH/gL that is stabilized by inter-protomer disulfide bonds;^61^ (2) a modified version of the multimerization domain from the C4b-binding protein from *Gallus gallus* (IMX313), which also forms a planar, ring-like structure stabilized by inter-protomer disulfide bonds capable of displaying seven copies of gH/gL;^62^ (3) *H. pylori* ferritin, which assembles into a 24-mer nanoparticle with octahedral symmetry and has previously been used to multimerize the EBV gp350 and gH/gL proteins;^43^^,^^63^ and (4) a secretion-optimized variant of a computationally designed, self-assembling 60-mer with icosahedral symmetry.^64^ The gH fusion proteins were co-expressed with gL using the Daedalus lentiviral expression system in HEK293 cells.^65^ The gH/gL fusion proteins were purified by affinity chromatography followed by size-exclusion chromatography (SEC). The average yields in mg/L of each purified gH/gL protein are provided in Table S1. The SEC elution profiles of the gH/gL fusion proteins were consistent with their expected size (Figure 1A; Table S2). The 4- and 7-mer constructs eluted earlier than the monomer. The gH/gL 60-mer eluted in the void volume as expected, while the gH/gL 24-mer eluted near the void volume. SEC coupled with multi-angle light scattering (SEC-MALS) revealed that the molecular weights of the particles were ∼540, ∼670, ∼4,420, and ∼7,400 kDa for the 4-, 7-, 24-, and 60-mer, respectively, which are close to their predicted nanoparticle sizes (Table S2). Bands corresponding to the expected sizes of the gH fusion proteins were identified by reducing SDS-PAGE (Figure 1B). Non-reducing SDS-PAGE revealed higher molecular weight complexes of the 4- and 7-mer consistent with the formation of inter-protomer disulfide bonds between the multimerization domain subunits (Figure 1C). These analyses also revealed a band corresponding to gL and demonstrated that the preparations were highly pure (Figures 1B and 1C).

The gH/gL nanoparticles were imaged using negative-stain electron microscopy (nsEM), which demonstrated that all particles were monodisperse and of the predicted size. Density corresponding to gH/gL emanating from the nanoparticle cores was apparent in 2D class averages of the 4-, 7-, and 24-mer (Figure 1D). Density corresponding to gH/gL was less clearly defined on the 60-mer particles, indicating conformational flexibility around the gH-I3 fusion junction.

To ensure that fusion to the multimerization domains did not alter the antigenicity of gH/gL, we measured the binding of several anti-gH/gL mAbs to each nanoparticle using an ELISA assay where biotinylated monomeric or gH/gL nanoparticles were captured on an ELISA plate coated with streptavidin. Of all the mAbs, AMMO1 binds with the highest affinity to monomeric gH/gL (Figure 1E).^55^ The AMMO1 epitope bridges domain I and domain II (D-I/D-II) and spans both gH and gL.^55^ CL40 has the second highest affinity (Figure 1E)^55^ and binds to an epitope spanning the D-II/D-III interface of gH.^54^ CL59 binds at the C terminus of gH on D-IV^54^ and has lower affinity than CL40 or AMMO1 (Figure 1E).^55^ E1D1 binds exclusively to gL and has the lowest affinity for the complex (Figure 1E).^55^^,^^56^

The mAbs maintained binding to each multimeric construct, and some showed significant improvements in binding to the nanoparticles (Figures 1E–1I). Despite showing the weakest binding of all the mAbs to the gH/gL monomer, E1D1 showed the strongest binding to the 7- and the 24-mer (Figures 1G and 1H). The E1D1 epitope is most distal to the multimerization domains and is therefore highly exposed on the nanoparticles. Moreover, the spacing of the E1D1 epitope may be optimally presented for bivalent engagement by the E1D1 mAb in some formats. In contrast, CL59 showed the weakest binding to all the gH/gL nanoparticles. CL59 binds closer to the C terminus of the gH ectodomain, which would be in close proximity to the nanoparticle core, potentially limiting exposure of the epitope (Figures 1F–1I). With the exception of E1D1, we did not observe a significant improvement in binding for most mAbs in the 60-mer format relative to the monomer in this assay.

### Immunogenicity of gH/gL nanoparticles

**Figure 2:**
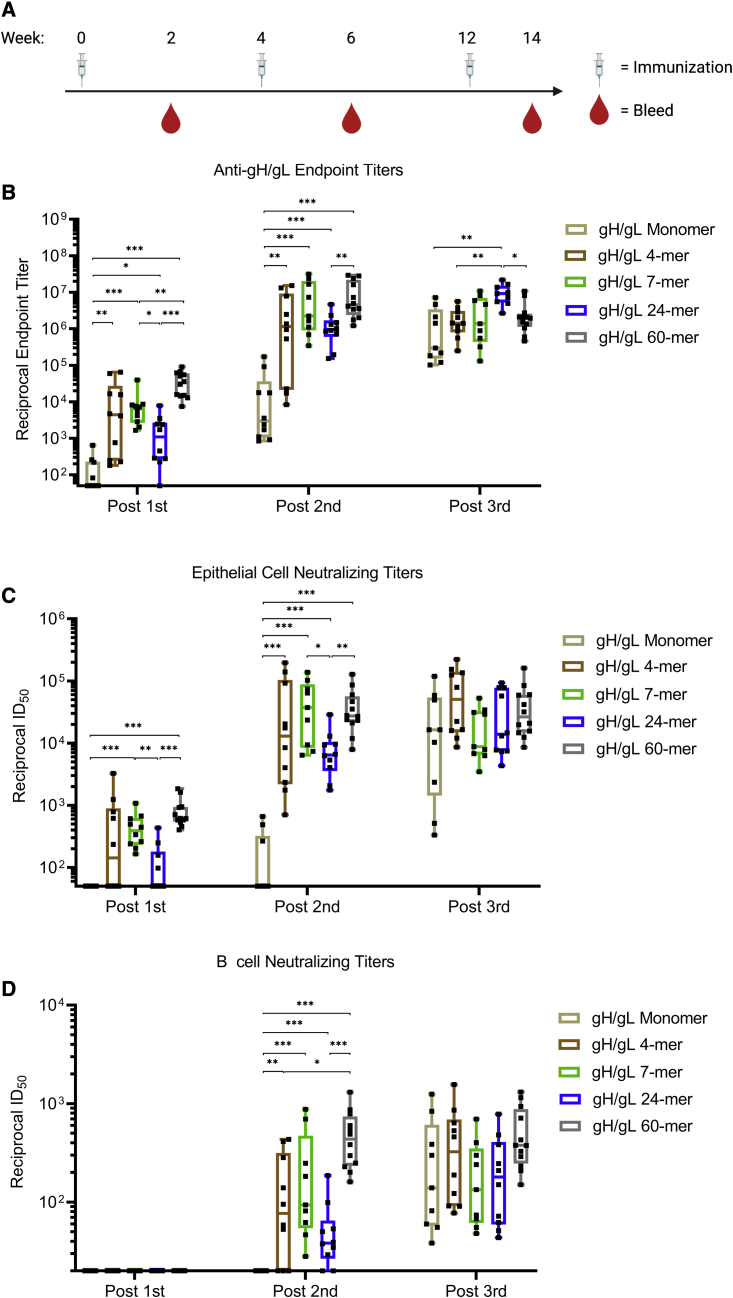
Immunogenicity of gH/gL nanoparticles (A) C57BL/6 mice (n = 10 mice for gH/gL monomer and 4-, 7-, and 24-mer, and n = 12 for gH/gL 60-mer) were immunized with monomeric gH/gL or multimeric gH/gL nanoparticles at weeks 0, 4, and 12. Blood was collected 2 weeks after each immunization. (B) Endpoint plasma binding titers to gH/gL were measured by ELISA. Each dot represents the reciprocal endpoint titer for an individual mouse measured in duplicate. Box and whisker plots represent the minimum, 25^th^ percentile, median, 75^th^ percentile, and maximum values. (C and D) The ability of plasma from individual mice to neutralize EBV infection of epithelial cells (C) or B cells (D). Each dot represents the reciprocal half-maximal inhibitory dilution (ID_50_) titer of an individual mouse. Plasma that did not achieve 50% neutralization at the lowest dilution tested (1:20) was assigned a value of 10. Box and whisker plots represent the minimum, 25^th^ percentile, median, 75^th^ percentile, and maximum values. Significant differences in B–D were determined using Mann-Whitney tests with Holm-adjusted p values (∗p < 0.05, ∗∗p < 0.01, ∗∗∗p < 0.001). See also Figures S1 and S2.

To assess the immunogenicity of the gH/gL nanoparticles, we immunized C57BL/6J mice with 5 μg of gH/gL monomer and 4-, 7-, 24-, or 60-mer formulated with adjuvant at weeks 0, 4, and 12. Plasma was collected 2 weeks after each immunization (Figure 2A). Endpoint binding titers to gH/gL were measured by ELISA (Figure 2B). After the first immunization, the median reciprocal binding titers in the gH/gL 4-, 7-, 24-, and 60-mer groups were higher than those in the monomer group. A second immunization boosted the binding titers in each group 200- to 1,000-fold. Again, the median titers in animals immunized with the gH/gL 4-, 7-, 24-, and 60-mer were higher than in those immunized with monomeric gH/gL.

A third immunization with the monomer boosted the gH/gL binding titers such that they were comparable to those elicited by the 4-, 7-, and 60-mer. A third immunization with the 24-mer also boosted the titers such that they were higher than the monomer 4- and 60-mer groups, while the third immunization with the other nanoparticles did not further boost the median binding titers (Figure 2B).

We next measured the ability of vaccine-elicited plasma to neutralize EBV infection of both B cells and epithelial cells. To monitor neutralization in epithelial cells, we used the SVKCR2 cell line that stably expresses CR2, which promotes cellular attachment of virions via gp350 improving the otherwise poor infectivity of epithelial cells *in vitro*.^66^ Neutralizing activity against epithelial cell infection was elicited 2 weeks after the first immunization in all groups that received multimeric, but not monomeric, gH/gL. The median reciprocal half-maximal inhibitory dilution (ID_50_) titers were significantly higher in the 60-mer group compared with the monomer and 24-mer groups (Figures 2C and S1). Additionally, median titers were significantly higher in the 7-mer group compared with the monomer and 24-mer groups.

The second immunization boosted median neutralizing titers by ∼10- to 100-fold in the epithelial cell infection assay. The median neutralizing titers were higher in all of the gH/gL-nanoparticle-immunized groups than they were in the monomer group (Figures 2C and S1). The epithelial cell neutralizing titers in the 7- and 60-mer were also higher than those elicited by the 24-mer. The third immunization with the gH/gL nanoparticles did not further boost epithelial cell neutralizing responses, while the third dose of monomeric gH/gL boosted titers to levels that were comparable with those in other groups.

None of the gH/gL antigens elicited antibodies that could neutralize EBV infection of B cells 2 weeks after the first immunization (Figures 2D and S2). Following the second immunization, neutralizing titers were present in plasma from all groups immunized with gH/gL nanoparticles but not in animals immunized with the monomer. Among the nanoparticle-immunized mice, the B cell neutralizing titers elicited by the 60-mer were higher than the 4- and 24-mer at this time point. Although the median B cell neutralizing titers elicited by the 60-mer (reciprocal ID_50_ = 436) were higher the 7-mer (reciprocal ID_50_ = 94), the difference was not statistically significant.

As was observed with the epithelial cell neutralizing titers, a third immunization with the gH/gL nanoparticles did not further boost B cell neutralizing responses, while a third dose of monomeric gH/gL boosted titers to levels that were comparable with those in other groups. In general, the neutralizing titers were about 10-fold lower against B cell infection compared with epithelial cell infection in all groups. From these analyses, we conclude that all gH/gL nanoparticles displayed superior immunogenicity compared with monomeric gH/gL after one or two immunizations and that a third immunization did not result in a significant titer boost.

### Plasma epitope mapping

**Figure 3:**
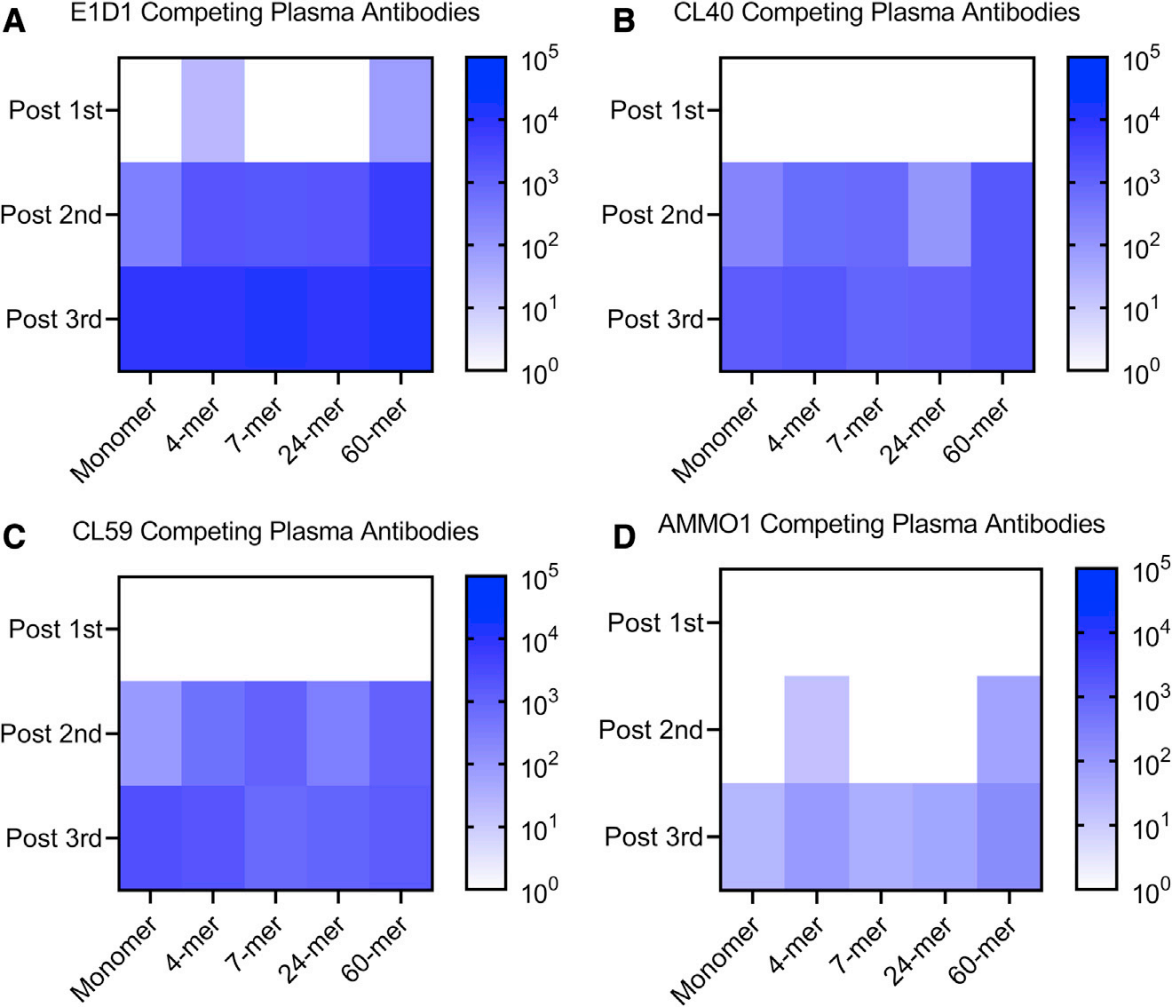
Plasma competition against monoclonal anti-gH/gL antibodies The ability of plasma pooled from groups of mice immunized with monomeric gH/gL or multimeric gH/gL nanoparticles to inhibit binding to a panel of anti-gH/gL antibodies to monomeric gH/gL was measured by ELISA. (A–D) The heatmap depicts the log reciprocal plasma dilution titers resulting in a 50% inhibition of (A) E1D1, (B) CL40, (C) CL59, or (D) AMMO1 antibodies at each time point. See Figure S3 for titration curves.

Each multimeric gH/gL nanoparticle tested here has a unique valency and geometry that differentially affects the exposure of certain epitopes bound by neutralizing anti-gH/gL mAbs (Figures 1D–1H). To test whether the nanoparticle format skewed the epitope specificity of vaccine-elicited antibodies from each construct, we assessed the ability of pooled immune plasma to compete with the E1D1, CL40, CL59, and AMMO1 mAbs for binding to monomeric gH/gL by ELISA (Figures 3A–3D).

Pooled plasma collected following one immunization with the gH/gL 4-mer and gH/gL 60-mer weakly inhibited E1D1 binding (Figures 3A and S3A). After the second immunization, plasma from all groups inhibited CL40, CL59, and E1D1 binding (Figures 3A–3C and S3B). Plasma antibodies that inhibited binding of these mAbs were further boosted following a third immunization in most groups. The only exception was that a third immunization with the 7-mer did not boost CL59-blocking plasma antibodies (Figures 3C and S3C).

Plasma antibodies capable of inhibiting AMMO1 binding were less common. Immune plasmas from the 4- and 60-mer groups weakly inhibited AMMO1 binding after two immunizations and were boosted following a third immunization (Figures 3D and S3C). All antigens elicited low titers of AMMO1-blocking antibodies following three immunizations. Among these, titers elicited by the 60-mer were highest at ∼1:150.

These experiments demonstrate that each gH/gL nanoparticle readily elicits antibodies that compete with E1D1 and that AMMO1-competing antibodies are rarer. This difference in competition could be attributed to the relative affinities of these mAbs for gH/gL (Figure 1E), or it could be due to the relative exposure of these epitopes on the nanoparticle.

**Figure 4:**
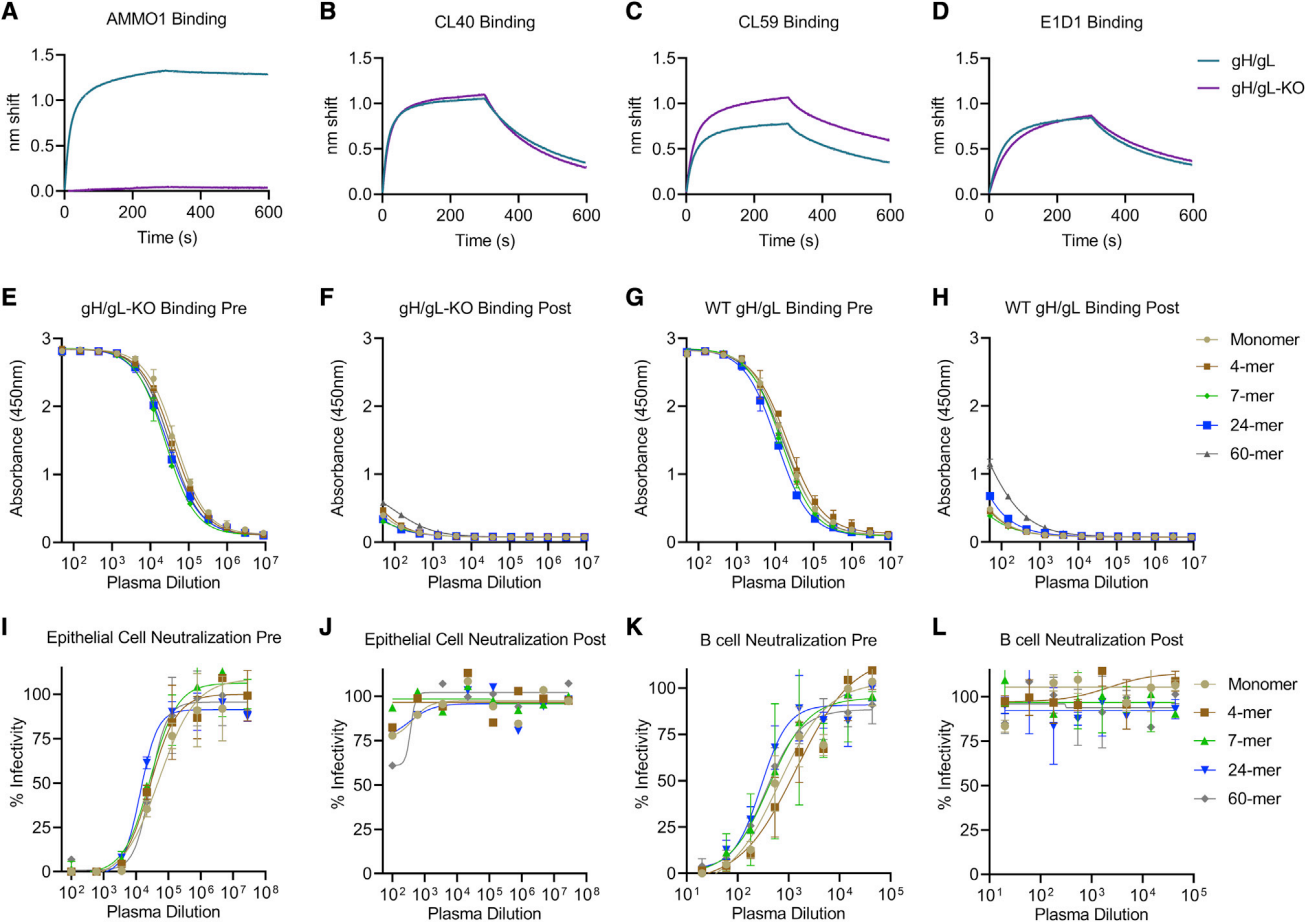
Depletion of AMMO1-KO-insensitive antibodies from pooled plasma (A–D) The binding of AMMO1 (A), CL40 (B), CL59 (C), and E1D1 (D) binding to gH/gL and gH/gL-KO (gH K73W,Y76A/gL) were measured using biolayer interferometry. (E–H) Antibodies were depleted from pooled plasma collected following three immunizations with gH/gL or gH/gL nanoparticles using gH/gL-KO conjugated magnetic beads. Pre- and post-depletion plasma samples were assayed for binding to gH/gL and gH/gL-KO by ELISA as indicated. Each data point represents mean, and error bars represent the standard deviation of two technical replicates. (I–L) The ability of plasma pre- and post-depletion to neutralize EBV infection was measured in B cells and epithelial cells. Each data point represents the mean, and error bars represent the standard deviation of two technical replicates.

Although the titers of AMMO1-competing antibodies in the plasma of mice immunized with gH/gL nanoparticles are low because the epitope bound by this mAb represents a critical site of vulnerability on gH/gL, we sought to assess the relative contribution of AMMO1-like antibodies to the plasma neutralizing activity of immunized mice. To achieve this, we developed an epitope-specific gH/gL probe and carried out plasma depletions. We previously identified two mutations, K73W and Y76A, that reduced binding of AMMO1 to cell-surface-expressed gH/gL.^55^ We expressed and purified a monomeric gH/gL ectodomain harboring these two mutations (herein called gH/gL-knockout [KO]), which completely ablated AMMO1 binding while maintaining binding to other gH/gL mAbs as measured by biolayer interferometry (BLI) (Figures 4A–4D).

Antibodies from pooled plasma collected from each group 2 weeks after the third immunization were depleted using immobilized gH/gL-KO. ELISA binding of depleted plasma to gH/gL-KO confirmed depletion of gH/gL-KO-specific antibodies (Figure 4, compare 4E and 4F). Depletion with gH/gL-KO also reduced binding to wild-type gH/gL (Figures 4G and 4H). The binding signal was slightly stronger for gH/gL relative to gH/gL-KO post-depletion (Figures 4H and 4F), suggesting that very few plasma antibodies are sensitive to the KO mutations in the serum. Although we cannot completely rule out the presence of antibodies that share the AMMO1 binding footprint but are insensitive to the KO mutations in this assay, these results are consistent with the mAb competition studies, which demonstrated that there are very few AMMO1-like antibodies in the plasma of immunized animals (Figures 3D and S3).

Depletion of gH/gL-KO-specific antibodies led to a complete loss of neutralizing titers in both the B cell and epithelial neutralization assays (Figures 4I–4L). Collectively, these data demonstrate that only a small portion of vaccine-elicited antibodies in each group target the AMMO1 epitope and that they do not make a measurable contribution to the plasma neutralizing activity.

### Passive transfer of nanoparticle-elicited gH/gL mAbs protects against lethal challenge in humanized mice

Immunocompromised mice engrafted with human hematopoietic stem cells develop human B cells that can become infected by EBV and are used as an *in vivo* model of EBV infection.^67^^,^^68^ This model has been used to evaluate the ability of monoclonal or polyclonal antibodies elicited by either vaccination or infection to protect against controlled viral challenge.^58^^,^^59^^,^^69^^,^^70^ Having established that gH/gL nanoparticles display superior immunogenicity, we sought to assess whether the antibodies they elicit confer protection against EBV challenge in this model.

To generate mice for these studies, non-obese diabetic [NOD] Rag1^-/-^, Il2rg^-/-^ mice were engrafted with mobilized huCD34+ hematopoietic stem cells, hereafter referred to as humanized mice. At 20 weeks post-transplant, 1 day prior to challenge, ∼10%–25% of peripheral blood mononuclear cells were human cells, of which ∼65%–80% were huCD19+ B cells (Figure S4). Since the humanized mouse model used here does not efficiently generate antibody responses to immunization,^71^ we opted to passively transfer purified antibodies elicited in wild-type C57BL/6 mice, which allowed us to directly evaluate the protective efficacy of the vaccine-elicited antibodies independent of other vaccine-induced immune responses.

To generate sufficient antibody for these experiments, C57BL/6J mice (n = 20) were immunized two times with 5 μg of the gH/gL 60-mer at weeks 0 and 4. This nanoparticle was selected because after two doses it consistently elicited high titers of antibodies that neutralize EBV infection of B cells, which are the primary targets of infection in humanized mice. As a comparator, we also immunized a group of mice with gH/gL monomer (n = 20). Two weeks after the second immunization, plasma were harvested and pooled, and total IgG was purified using protein A/G resin. As a control, IgG was purified from unimmunized C57BL/6J mice (n = 20).

**Figure 5:**
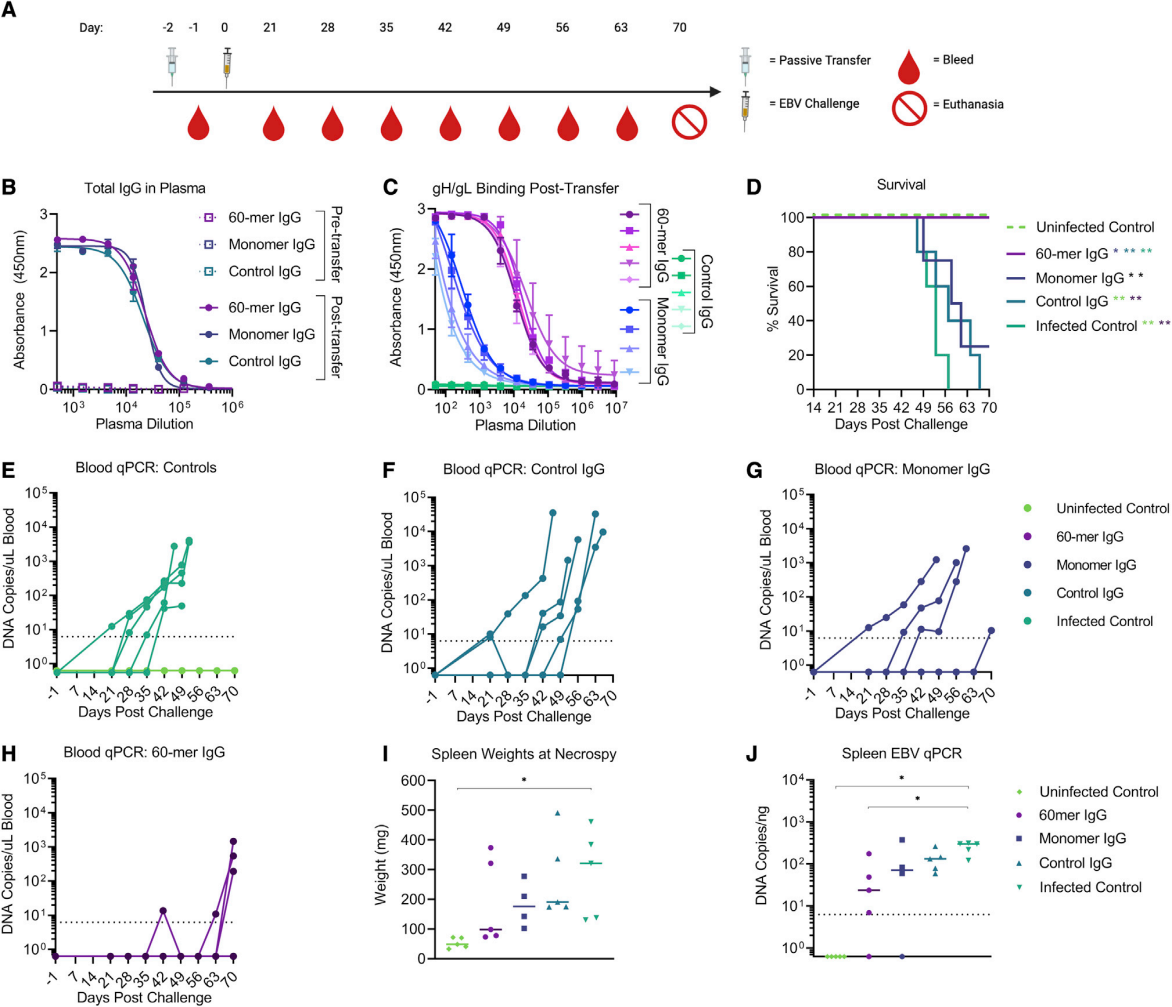
gH/gL-nanoparticle-elicited antibodies protect humanized mice from lethal EBV challenge C57 BL6 mice were immunized with either monomeric or gH/gL 60-mer (n = 20 per group) at weeks 0 and 4. Blood was collected by cardiac puncture at week 6 and pooled, and the serum IgG was purified. (A) 0.5 mg of total IgG from monomer (n = 4) or 60-mer (n = 5) immunized mice was administered to humanized mice. A control group of mice received 0.5 mg total IgG purified from naive C57 BL6 mice (n = 5). (B) Total IgG was measured in pooled plasma from each group collected 3 days prior to and 1 day after IgG transfer. Each data point represents mean, and error bars represent the standard deviation of two technical replicates. (C) Anti-gH/gL IgG antibodies from plasma collected from individual humanized mice 1 day after transfer was measured by ELISA as indicated. Each data point represents mean, and error bars represent the standard deviation of two technical replicates. (D) Survival of humanized mice that received IgG purified from the indicated groups was monitored over a 70 day period following EBV challenge. An infected control group (n = 5) did not receive IgG prior to challenge, and an uninfected control group (n = 5) did not receive IgG or viral challenge. Significant differences in the survival data were determined using log rank tests (∗p < 0.05, ∗∗p < 0.01). (E–H) Viral DNA was quantified in the peripheral blood of infected and uninfected control (E), control IgG (F), monomer IgG (G), and 60-mer IgG (H) groups collected at the indicated time points via qPCR. Each series of connected dots represents an individual mouse at each time point analyzed, and the dashed line represents the limit of detection. (I) At necropsy, spleens were harvested and weighed. Each dot represents an individual mouse, and the bar represents the median weight in milligrams. Significant differences in spleen weight were determined using Mann-Whitney tests with Holm-adjusted p values (∗p < 0.05). Photographs of individual spleens are shown in Figure S5. (J) Viral DNA copy number was quantified in splenic DNA extracts at necropsy. Each dot represents an individual mouse, the bar represents the median copy number, and the dashed line indicates the limit of detection. Significant differences in viral DNA copy number were determined using Mann-Whitney tests with Holm-adjusted p values (∗p < 0.05). See also Figures S4 and S5.

IgG from each group were delivered to humanized mice (n = 4-5 mice/group) via intraperitoneal injection 2 days prior to challenge at a dose of 500 μg IgG/mouse (Figure 5A). Total IgG measured in pooled plasma collected 2 days prior to and 1 day after transfer confirmed that that the mice in each group received comparable levels of total IgG (Figure 5B). However, the levels of anti-gH/gL antibodies were higher in the mice that received IgG from 60-mer-immunized animals compared with those that received IgG from animals immunized with the monomer (Figure 5C), consistent with the superior immunogenicity of the gH/gL nanoparticle (Figure 2B). Plasma from animals that received IgG from unimmunized animals (control IgG) did not display any binding activity to gH/gL (Figure 5C). Two days after IgG transfer (day 0), each mouse was challenged via retro-orbital injection with 33,000 Raji infectious units (RIUs) of EBV (Figure 5A). We also included an infected control group that did not receive antibody pre-treatment and an uninfected control group that received neither antibody pre-treatment nor EBV challenge.

Following challenge, the mice were weighed three times a week and monitored for general health over the course of 10 weeks. Blood samples were collected weekly beginning at 3 weeks post-challenge, and spleens were harvested from each mouse at the day 70 endpoint or earlier if they met euthanasia criteria. Upon completion of the study, 100% of the animals in the uninfected control and 60-mer treatment groups survived (Figure 5D). In contrast, 100% of the animals in the infected control and control IgG treatment groups succumbed to infection by 56 and 66 days post-challenge, respectively. 75% of mice in the monomer treatment group did not survive beyond day 60, and only one animal survived the entire 70 days (Figure 5D). The survival rate of mice in the uninfected control group and 60-mer treatment group were significantly higher than all other groups (Figure 5D).

EBV DNA was not detected in the blood or spleen of the uninfected control group throughout the duration of the experiment (Figures 5E and 5J). In contrast, 100% of mice in the control IgG treatment group (Figure 5F) and 100% of mice in the infected control group were viremic as early as 21 days post-challenge (Figure 5E). In the monomer IgG group, EBV DNA was detected in the blood of 100% of mice (Figure 5G). In the 60-mer IgG group, EBV DNA was undetectable in blood of 40% of mice at any time point tested. One mouse was viremic at weeks 7 and 10, another at weeks 9 and 10, and a third at week 10 (Figure 5H).

A rapid decline in peripheral CD19^+^ B cells (Figure S4A) accompanied by an increase in peripheral CD8^+^ T cells (Figure S4B) was observed in the infected control mice and in mice that received control IgG or IgG elicited by the monomer, approximately 1 month post-challenge. This phenotype is consistent with high-dose EBV challenge^72^ and with T cell-mediated killing of infected B cells.^73^ A more gradual decline in B cell frequencies and increase in CD8^+^ T cell frequencies was observed in the uninfected control mice and in three of the mice that received IgG elicited by the gH/gL 60-mer (Figures S4A and S4B).

At necropsy, spleens from animals in the infected control group were significantly heavier than those in the uninfected control group (Figure 5I) and had visible splenic tumors (Figure S5). Spleens from two of the viremic mice in the 60-mer IgG group were about 3 times heavier than the other three mice in the 60-mer IgG group, and 3 of 5 spleens had visible tumors (Figure S5). Three of the spleens were heavier in the monomer group relative to the uninfected control, while the fourth was comparable with the uninfected controls and 3 of 5 of the 60-mer IgG-treated animals (Figure 5I). All mice in the monomer IgG group had visible splenic tumors (Figure S5). Viral DNA was not detected in the spleens of all animals in the uninfected control group and from the spleens from one animal in each of the 60-mer IgG and monomer IgG groups (Figure 5J), but it was detected in the spleens of all remaining mice (Figure 5J).

Collectively, these data demonstrate that multivalent display of gH/gL elicits higher titers of neutralizing antibodies that protect against lethal EBV challenge in a humanized mouse model. However, they do not confer sterilizing immunity.

## Discussion

A safe and effective vaccine could alleviate the global disease burden resulting from EBV infection. Here, we developed several multimeric vaccine candidates derived from the gH/gL ectodomain and evaluated their ability to elicit antibodies capable of neutralizing EBV infection of both B cells and epithelial cells in mice and demonstrated that a computationally designed nanoparticle displaying 60 copies of gH/gL elicited antibodies capable of protecting against high-dose, lethal challenge in a humanized mouse infection model.

Antigen multimerization has been used to improve the immunogenicity of subunit vaccines against several pathogens including malaria, HIV-1, respiratory syncytial virus (RSV), severe acute respiratory syndrome coronavirus 2 (SARS-CoV-2), influenza^74^, ^75^, ^76^, ^77^, ^78^, ^79^, ^80^, ^81^ and EBV.^43^^,^^60^^,^^69^ Multimerization can enhance the immunogenicity of subunit vaccines through several mechanisms including more efficient B cell receptor cross linking, triggering of innate B cell responses, lymph node trafficking, and enhanced major histocompatibility complex (MHC) class II antigen presentation.^82^, ^83^, ^84^ In general, we observed that mAb binding as measured by ELISA was generally improved by multimerization (Figures 1E–1I); however, this was not always the case, as the binding of most mAbs showed comparable binding with monomeric and 60-meric gH/gL. This discrepancy may be related to the efficiency of antigen biotinylation and/or capture on the ELISA plate. Nevertheless, nanoparticle display resulted in a significant improvement in immunogenicity (Figure 2).

Previous studies have shown that antigen valency correlates with B cell activation, germinal center recruitment, and B cell differentiation as well as serum binding and neutralizing titers.^77^^,^^85^^,^^86^ Although nanoparticles displaying gH/gL exhibited superior immunogenicity compared with monomeric gH/gL, we did not observe a strict correlation between antigen valency and binding or neutralizing titers. The differences in the ability of these antigens to elicit neutralizing antibodies could be linked to nanoparticle stability *in vivo* or T cell help directed at MHC class II-restricted epitopes that differ between the nanoparticle scaffolds.^87^

Because of its ability to potently neutralize infection of both cell types, the overlapping epitope targeted by AMMO1 and 769B10 represents a critical site of vulnerability on EBV.^43^^,^^55^ Despite readily eliciting antibodies targeting several other epitopes on gH/gL, our analysis indicates that the AMMO1 epitope is subdominant in the context of immunization. Despite this, immune plasma from gH/gL 60-mer immunized mice was protective *in vivo*. Thus, relative to monomeric gH/gL, the gH/gL 60-mer may have elicited high titers of less potent anti-gH/gL antibodies like CL40 and CL59. Alternatively, the immunogens may have elicited antibodies targeting other potently neutralizing epitopes on gH/gL such as the one defined by the recently identified 1D8 mAb or other yet-to-be-identified epitopes.^58^ Gaining a better understanding of the epitopes on gH/gL that are targeted by neutralizing and non-neutralizing antibodies elicited by natural infection or immunization through the isolation and characterization of mAbs would enable rational gH/gL vaccine design that could further enhance neutralizing titers when combined with multimeric antigen display. For example, immunogen design strategies could be employed to immunofocus the antibody response to potently neutralizing gH/gL epitopes.

The majority of humanized mice that received IgG elicited by monomeric gH/gL did not survive EBV challenge. Similarly, passive transfer of sera from rabbits immunized with monomeric gH/gL conferred partial protection from lethal EBV challenge in humanized mice.^69^ Since EBV infection in humanized mice is restricted to human B cells,^67^^,^^68^ the observed lack of protection by IgG raised against monomeric gH/gL in our study is most likely due to the inability of this antigen to elicit antibodies that neutralize infection of B cells following two immunizations. In contrast, IgG purified from animals immunized with the gH/gL 60-mer prevented death within a 10 week window following high-dose EBV challenge, demonstrating that multivalent display substantially improves the quality of vaccine-elicited anti-gH/gL antibodies. We note that 3/5 animals in this group were viremic and had obvious tumors at the study endpoint and that a 4^th^ had trace amounts of viral DNA in the spleen, thus sterilizing immunity was not achieved in this model, and it is possible that some of the animals may have succumbed to infection if observed for a longer period.

In sum, we demonstrate that multivalent display of EBV gH/gL markedly enhanced immunogenicity in mice and that a computationally designed 60-mer nanoparticle elicited antibodies that protected against lethal challenge in a humanized mouse infection model. These results underscore the importance that vaccine-elicited antibodies against gH/gL can play in preventing EBV infection and highlight the utility of cutting-edge vaccine approaches in the development of vaccines against this important cancer-associated pathogen.

### Limitations of the study

Although we only evaluated the ability of antibodies elicited by the 60-mer in the humanized mouse challenge studies, the other nanoparticles developed here and elsewhere^43^^,^^69^ have potential for clinical development and additional *in vivo* comparisons and manufacturing feasibility studies are warranted.

Both B cells and epithelial cells are present in the oropharynx, thus antibodies that can neutralize infection of both types of cells are an important consideration for EBV vaccine development.^88^ The gH/gL constructs developed here and by others^43^^,^^69^ consistently elicit higher epithelial cell neutralizing titers compared with B cell neutralizing titers. Since murine epithelial cells are not susceptible to infection and oral transmission is not possible in humanized mice,^68^^,^^89^ this challenge model may underestimate the relative importance of antibodies capable of neutralizing infection of this cell type. Moreover, it is not understood how an intravenous dose of virus in humanized mice compares with the innoculum in a natural oral exposure. Thus, the evaluation of a multivalent gH/gL vaccine to prevent rhesus lymphocryptovirus infection of macaques, where oral transmission is the natural route of infection,^90^ should more accurately predict its ability to protect humans against EBV.

## STAR★Methods

### Key resources table

**Table:** 

REAGENT or RESOURCE	SOURCE	IDENTIFIER
**Antibodies**		

Recombinant AMMO1	Snijder et al., 2018	N/A
Recombinant CL40 with human constant regions	Singh et al., 2020	N/A
Recombinant CL59 with human constant regions	Singh et al., 2020	N/A
Recombinant E1D1 with human constant regions	This study	N/A
hCD20- BV786	BD Biosciences	Cat# 743611; RRID:AB_2741622
hCD45-FITC	ThermoFisher	Cat# 5010066; RRID:AB_1907394
mCD45-APC	Biolegend	Cat# 103112; RRID:AB_312977
hCD33-PE	BD Biosciences	Cat# 555450; RRID:AB_395843
hCD19- BV711	Biolegend	Cat# 302246; RRID:AB_2562065
hCD4- AF700	ThermoFisher	Cat# 56-0048-82; RRID:AB_657741
hCD8-BV421	BD Biosciences	Cat# 562429; RRID:AB_11153676
mCD16/32	Biolegend	Cat# 101302; RRID:AB_312801
Rabbit anti-His tag	Sigma Aldrich	Cat# SAB5600227
Goat anti-mouse IgG-HRP	SouthernBiotech	Cat# 1033–05; RRID:AB_2737432
Goat anti-human IgG-HRP	SouthernBiotech	Cat# 2010–05; RRID:AB_2795564
Goat anti-human IgG-HRP	Jackson ImmunoResearch	Cat# 115-035-008; RRID:AB_2313585
Goat anti-human IgG	SouthernBiotech	Cat# 1030–01; RRID:AB_2794290
Goat Anti-Mouse IgG, Human ads-HRP	SouthernBiotech	Cat# 1030–05; RRID:AB_2619742

**Bacterial and virus strains**		

EBV B95.8/F	Delecluse et al., 1998	N/A
EBV AKTA-GFP	Molesworth et al., 2000	N/A

**Biological samples**		

human CD34-enriched PBSCs	Co-operative Center for Excellence in Hematology, Fred Hutchinson Cancer Research Center	N/A

**Chemicals, peptides, and recombinant proteins**		

Platinum Super-Fi II DNA polymerase	ThermoFisher	Cat# 12361010
In-fusion HD cloning kit	Takara Bio	Cat# 638920
Polybrene	Millipore Sigma	Cat# TR-1003-G
293Freestyle Media	ThermoFisher	Cat# 12338018
Phosphate Buffered Saline	Corning	Cat# 21-040-CM
Protein A Agarose	GoldBio	Cat# P-400-5
Pierce IgG Elution Buffer	ThermoFisher	Cat# 21004
Ni-NTA resin	ThermoFisher	Cat# 88221
Galanthus Nivalis Lectin Agarose	Vector Laboratories	Cat# AL-1243-5
disuccinimidyl suberate	Thermofisher	Cat# 21555
synthetic lipid A in squalene emulsion SLA-SE	Infectious Disease Research Institute	N/A
Sigma Adjuvant System	Sigma Aldrich	Cat# S6322
Pierce Protein A/G Binding Buffer	ThermoFisher	Cat# 54200
Pierce Protein A/G resin	ThermoFisher	Cat# 20422
GeneJuice Transfection Reagent	Sigma Aldrich	Cat# 70967
293-Free Transfection Reagent	Millipore Sigma	Cat# 72181
0.25% trypsin	ThermoFisher	Cat# 25200056
10% Formalin	Millipore Sigma	Cat# HT501128
eBioscience Fixable Viability Dye eFluor 506	ThermoFisher	Cat# 65-0866-18
SureBlue Reserve TMB Microwell Peroxidase substrate	SeraCare	Cat# 5120–0081
EZ-Link NHS-PEG4-Biotin Kit	ThermoFisher	Cat# 21330
Zeba Spin Desalting Columns 40K MWCO	ThermoFisher	Cat# 87766
NeutrAvidin Protein	ThermoFisher	Cat# 31000
Streptavidin Magnetic Beads	New England BioLabs	Cat #S1420S

**Critical commercial assays**		

DNeasy Blood & Tissue Kit	Qiagen	Cat# 69504
2×QuantiTect Probe PCR Master Mix	Qiagen	Cat# 204343
Anti-Human Fc Capture (AHC) Biosensors	Sartorius	Cat# 18–5060
QuickChange XL II Kit	Agilent	Cat# 200521
TaqMan Copy Number Reference Assay, human, RNase P	Thermofisher	Cat# 4403326

**Experimental models: cell lines**		

Raji	ATCC	CCL-86
293-6E cells	National Research Council, Canada	RRID:CVCL_HF20
293–2089	Delecluse et al., 1998	N/A
SVKCR2	Li et al., 1992b	RRID:CVCL_YD67
293T		RRID:CVCL_0063
AKATA-BX1-GFP	Molesworth et al., 2000)	

**Experimental models: Organisms/strains**		

NOD-*scid* Il2rg^null^ mice	The Jackson Laboratory	Stock No: 005557

**Oligonucleotides**		

Forward primer specific for EBV BALF5 gene: CCCTGTTTATCCGATGGAATG	Kimura et al., 1999	N/A
Reverse primer specific for EBV BALF5 gene: CGGAAGCCCTCTGGACTTC	Kimura et al., 1999	N/A
FAM-labeled probe specific for EBV BALF5 gene: CGCATTTCTCGTGCGTGTACACC	Kimura et al., 1999	N/A

**Recombinant DNA**		

BALF5 Target DNA for standard: ACCGAGACCCGGCAGGGGGTCCTGCGGTC GAAGGTGCTGGCCTTGAGGGCGCTGAGGA CTGCAAACTCCACGTCCAGACCCTGAGGC GCGCTGGCGTAGAAGTAGGCCTGCTGCCC AAACACGTTCACACACACGCTGGCCCCAT CGGCCTTGCGCCGGCCCAGTAGCTTGAT GACGATGCCACATGGCACCACATACCCCT GTTTATCCGATGGAATGACGGCGCATTTCT CGTGCGTGTACACCGTCTCGAGTATGTCG TAGACATGGAAGTCCAGAGGGCTTCCGTG GGTGTCTGCCTCCGGCCTTGCCGTGCCCT CTTGGGCACGCTGGCGCCACCACATGCCC TTTCCATCCTCGTCACCCCCCACCACCGTC AGGGAGTCTTGGTAGAAGCACAGGGGGGG CTGAGGCCCCCGCACATCCACCACCCCTGC GGCGCCTGGTGTCTGGAAACACTTGGGAAT GAGACGCAGGTACTCCTTGTCAGGCTTTTTC	Singh et al., 2020	Custom Synthesis Integrated DNA technologies
p2670	Delecluse et al., 1998	N/A
p509	Neuhierl et al., 2002	N/A
pTT3-gH-HIS-AVI	Snijder et al., 2018	N/A
pTT3-gL	Snijder et al., 2018	N/A
pTT3-gH	This Study	N/A
pTT3-gH-K73W-Y76A	This Study	N/A
pTT3-gH-IMX313	This Study	N/A
pTT3-gH-ferritin	This Study	N/A
pCVL-UCOE0.7-SFFV-muScn-IRES-GFP	Bandaranayake et al., 2011	N/A
pCVL-UCOE0.7-SFFV-gH-C153T-IMX313-IRES-GFP	This Study	N/A
pCVL-UCOE0.7-SFFV-gH-ferritin-IRES-GFP	This Study	N/A
pCVL-UCOE0.7-SFFV-gH--C153T-I3-IRES-GFP	This Study	N/A
pCVL-UCOE0.7-SFFV-gH-C153T-cTRP(6)ss-IRES-GFP	This Study	N/A
pCVL-UCOE0.7-SFFV-muScn-IRES-RFP	Bandaranayake et al., 2011	N/A
pCVL-UCOE0.7-SFFV-gL-IRES-RFP	This Study	N/A
psPAX2	Addgene	#12260
pMD2.G	Addgene	#12259

**Software and algorithms**		

QuantaSoft™ Analysis Software	Bio-Rad	N/A
Prism 9.2.0 or later software package	Graph Pad Software	N/A
Leginon	Suloway et al., 2005	N/A
cryoSPARC	Punjani et al., 2017	N/A
CTFFIND	Rohou and Grigorieff, 2015	N/A
Appion	Lander et al., 2009	N/A
CTFFIND4	Mindell and Grigorieff, 2003	N/A
DoG picker	Voss et al., 2009	N/A
EMAN 1.9	Ludtke et al., 1999	N/A
RELION/2.1	Kimanius et al., 2016; Scheres, 2012a;	N/A
CryoSPARC	Punjani et al., 2017	N/A
CTFFIND4	Mindell and Grigorieff, 2003	N/A
ChimeraX	Pettersen et al., 2021	N/A
EPU	ThermoFisher	N/A
ASTRA	Wyatt Technologies	N/A

**Other**		

QuantStudio 7 Flex Real-Time PCR System	Applied Biosystems	N/A
HiLoad 16/600 Superdex 200 pg	Millipore Sigma	Cat# GE28-9893-35
Superose 6 Increase 10/300 G	Millipore Sigma	Cat# GE29-0915-96
Hi-Trap Q HP	Millipore Sigma	Cat# GE29-0513-25
C1000 Touch Thermal Cycler	Bio-Rad	N/A
NGC FPLC	Bio-Rad	N/A
SpectraMax M2 plate reader	Molecular Devices	N/A
BD LSRII cytometer	BD Biosciences	N/A
Guava HT cytometer	Luminex	N/A
Octet Red 96E	Sartorius	N/A
1260 High-Performance Liquid Chromatography System	Agilent	N/A
Heleos multi-angle light scattering detector	Wyatt Technology	N/A
tREX refractive index detector	Wyatt Technology	N/A

### Resource availability

#### Lead contact

Requests for resources and reagents should be directed to and will be fulfilled by Andrew T. McGuire (amcguire@fredhutch.org).

#### Materials availability

All materials generated herein are available upon request under an MTA from the corresponding author (amcguire@fredhutch.org). The pTT3 vectors are used under license from the National Research Council of Canada. The UCOE element in the pCVL- UCOE0.7-SFFV based vectors is used under license from Millipore.

### Experimental model and subject details

#### Mice

Comparative immunogenicity studies and the elicitation of polyclonal antibodies for passive transfer studies were performed in an equal mix of male and female C57BL/6 mice between 7 and 10 weeks of age. C57BL/6 mice were purchased from Jackson Labs and housed in a specific pathogen-free facility at the Fred Hutchinson Cancer Research Center.

6–7 week old NOD-scid Il2rg^null^ (NSG) NSG mice were irradiated with 275 Roentgen and then engrafted with 1×10^6^ CD34-enriched PBSCs obtained from granulocyte colony-stimulating factor mobilized healthy donors by intravenous injection (herein called humanized mice). All humanized mice used in this study were female and were engrafted with CD34-enriched PBSCs from the same female donor. Humanized mice were purchased from the Co-operative Center for Excellence in Hematology, Fred Hutchinson Cancer Research Center. Prior to EBV challenge, humanized mice werehoused in a specific pathogen-free facility and after EBV challenge the animals were housed in an animal basic safety level 2 facility at the Fred Hutchinson Cancer Research Center. All mice used in our studies were housed with free access to food and water with a 12:12 light:dark cycle. The animal facilities are accredited by the Association for Assessment and Accreditation of Laboratory Animal Care. Mice were handled in accordance with the NIH Guide for the Care and Use of Laboratory Animals. All animal experiments were approved by the Fred Hutch Institutional Animal Care and Use Committee and Institutional Review Board.

#### Cell lines

All cell lines were incubated at 37°C in the presence of 5% CO2 and were not tested for mycoplasma contamination. Raji cells (human male) were maintained in RMPI + 10% FBS, 2 mM L-glutamine, 100 U/mL penicillin, and 100 μg/mL streptomycin (cRPMI). 293–2089 cells (human female) were grown in cRPMI containing 100 μg/mL hygromycin.^91^ AKATA (human female) B cells harboring EBV in which the thymidine kinase gene has been replaced with a neomycin and GFP cassette virus (AKATA-GFP) were grown in cRPMI containing 350 μg/mL G418.^48^ SVKCR2 cells (human male) were grown in DMEM containing 10% cosmic calf serum, 2 mM L-glutamine, 100 U/mL penicillin, 100 μg/mL streptomycin, 10 ng/mL cholera toxin and 400 μg/mL G418.^66^ 293-6E (human female, RRID:CVCL_HF20) and 293T cells (human female, RRID:CVCL_0063) cells were maintained in Freestyle 293 media with gentle shaking.

### Method details

#### Plasmids

pTT3 plasmids containing cDNA encoding gH (AA 19–679, GenBank: AFY97969.1) with a C-terminal His and Avi tag (pTT3-gH-HIS-AVI), and gL AA 24–137 GenBank: AFY97944.1 (pTT3-gL) with a TPA leader peptide have been previously described^55^. Site directed mutagenesis was used to introduce stop codons into gH between the His and Avi tags to produce an expression plasmid without the Avi tag, or 5′ of the His tag to produce an expression plasmid with no tags (pTT3-gH). The K73W and Y76A mutations gH (herein called gH/gL-KO), were introduced into pTT3-gH-His using the QuickChange XL II Kit according to manufacturer’s instructions.

To create a 7-mer gH expression construct, cDNA encoding a modified version of the C4b-BP protein (IMX313)^62^ followed by a stop codon was synthesized and cloned in-frame with the gH ectodomain in pTT3-gH-His-Avi, replacing the His and Avi tags to create pTT3-gH-IMX313. To create a 24-meric gH expression construct, cDNA encoding the gH ectodomain was amplified by PCR with primers that introduced an *Eco*RI site at the 5′ end followed by a (G_4_S)_2_ linker and finally a *Bam*HI site at the 3′ end. The PCR amplicon was cloned into pTT3-426cTM4ΔV1-3-ferritin^92^ (a kind gift from Dr. Leonidas Stamatatos) replacing the HIV-1 Env gene fused to *H. pylori* ferritin^63^ to create pTT3-gH-ferritin.

cDNA encoding gH-IMX313 and gH-ferritin were amplified by PCR, and then cloned into the *Xho*I and *Bam*HI restriction sites of pCVL-UCOE0.7-SFFV-muScn-IRES-GFP^65^ replacing the muSCN cDNA, to create pCVL-UCOE0.7-SFFV-gH-IMX313-IRES-GFP and pCVL-UCOE0.7-SFFV-gH-ferritin-IRES-GFP. A C153T mutation which replaces an unpaired cysteine in gH was added to pCVL- UCOE0.7-SFFV-gH-IMX313-IRES-GFP using the QuickChange XL II Kit according to manufacturer’s instructions.

pCVL-UCOE0.7-SFFV-gH-I3-C153T-IRES-GFP was created by synthesizing a g-block encoding a modified version of I3-01 (^64^ and J.Y.W. manuscript in preparation) with homology to the 3′ end of the gH ectodomain at the 5′ end of the g block and homology to the downstream IRES region at the 3′ end of the g block. The plasmid backbone was amplified from pCVL-UCOE0.7-SFFV-gH-IMX313-IRES-GFP using a reverse primer that annealed to the 3′ end of the gH cDNA (containing the C153T mutation) and a forward primer that annealed to the 5′ end of the IRES and Platinum Super-Fi II DNA polymerase. The g block and linearized plasmid backbone were ligated together using the In-fusion HD cloning kit.

The tetramerization domain from cTRP24_6_SS^61^ was amplified by PCR using primers that added homology to the 3′ end of the gH ectodomain at the 5′ end homology to the downstream IRES region at the 3′ of the amplicon. The amplicon was ligated to the PCR linearized plasmid backbone described above using the In-fusion HD cloning kit to create from pCVL-UCOE0.7-SFFV-gH-C153T-cTRP(6)ss-IRES-GFP.

cDNA encoding gL was amplified by PCR, and then cloned into the *Xho*I and *Bam*HI restriction sites of pCVL-UCOE0.7-SFFV-muScn-IRES-RFP^65^ replacing the muSCN cDNA, to create pCVL-UCOE0.7-SFFV-gL-IRES-RFP.

The sequences of all plasmids were confirmed by Sanger sequencing.

#### Lentiviral production

5.46 μg of psPAX2, 2.73 μg of pMD2.G (both gifts from Didier Trono), and 11.05 μg of each pCVL-derived gH plasmid were mixed in 1.56mL PBS followed by 39 μL of 293-Free Transfection Reagent. The transfection mix was gently agitated, incubated at room temperature for 15 min, and added dropwise to 13 mL of suspension-adapted 293T cells at 2 × 10^6^ cells/mL in a 125 mL flask. After 24 h, an additional 15 mL of 293 Freestyle media containing 15 μg of valproic acid was added to the cell culture. After another 48 h, the cell culture was centrifuged at 1000 × *g* for 3 min, the supernatant was passed through a 0.44 μm filter, aliquoted, and stored at −80°C.

#### Lentiviral transduction

Polybrene was added to 10 mL of 293-6E cells at 1×10^6^ cells/mL to a final concentration of 2 μg/mL in addition to 2–3 mL of supernatant containing lentiviral particles harboring the various gH and gL expression constructs. 24 h following transduction, 15 mL of 293Freestyle media was added to the culture. A Guava easyCyte Flow Cytometer was used to monitor gH (GFP^+^) and gL (RFP^+^) transduction efficiency 72 h after transduction. Transduced cultures were expanded to a total volume of 1 L and cultured until cell viability declined to ∼80%. The transduced cell cultures were centrifuged at 4000 × *g* for 10 min to pellet cells. The supernatant was further clarified by passing through a 0.22 μm filter.

#### Purification of untagged monomeric gH/gL

Clarified cell supernatant was adjusted to pH 5.5–6 using 2 M acetic acid. The clarified cell supernatant was incubated with CaptoMMC resin, pre-equilibrated with 30 mM sodium acetate, 50 mM NaCl, pH 5.5 (MMC binding buffer), then washed with 10 column volumes of MMC binding buffer and then eluted with 10 column volumes of 50 mM sodium acetate, 1 M ammonium chloride, pH 7.4. The protein elute was collected and concentrated using an Amicon Ultra-4 Centrifugal Filter Unit, and further purified via size exclusion chromatography (SEC) on a HiLoad 16/600 Superdex 200 pg column with 10 mM Tris, 50 mM NaCl, pH 7.4 as the mobile phase. The protein was further purified by anion exchange chromatography using a HiTrap Q HP column pre-equilibrated with 10 mM Tris, 50 mM NaCl, pH 7.4. The column was washed with 7% elution buffer (10 mM Tris, 1 M NaCl, pH 7.4) until the absorbance at 280nm (A_280_) achieved a stable baseline. gH/gL was eluted over a linear gradient from 7% to 25% elution buffer over 20 column volumes. The eluted protein was further purified by SEC with PBS (PBS) as the mobile phase on the Superose six Increase 10/300 GL. Fractions were analyzed by SDS-PAGE to identify those containing gH/gL >95% purity based on Coomassie blue staining. The purified protein was aliquoted, flash frozen in liquid nitrogen and stored long term at −80°C.

#### Purification of polyhistidine tagged proteins gH/gL His, gH/gL-KO, and gH/gL-cTRP(6)ss

Clarified cell supernatant was adjusted to a final concentration of 10 mM imidazole and 500 mM NaCl and then incubated with HisPur Ni-NTA resin pre-equilibrated with 10 mM Tris, 500 mM NaCl, 10 mM imidazole, 0.02% azide, pH 7.1 (Ni-NTA binding buffer). The column was then washed with 10 column volumes of Ni-NTA binding buffer and eluted using 10 mM Tris, 500 mM NaCl, 500 mM imidazole, pH 8.0. The NiNTA eluate was subsequently purified by SEC using a Superdex 200 column with PBS as the mobile phase. Purified protein was aliquoted flash frozen in liquid nitrogen and stored at −80°C.

#### Purification of gH/gL-4-mer (gH/gL-IMX313) and gH/gL-ferritin

Clarified cell supernatant was adjusted to a final concentration of 100 mM NaCl and then incubated with Galanthus Nivalis Lectin Agarose, washed with 10 column volumes of 20 mM Tris, 100 mM NaCl, 1 mM EDTA, pH 7.4 and eluted with 20mM Tris, 100 mM NaCl, 1 mM EDTA, 1M methylmannopyranoside, pH 7.4. The eluted protein was further purified by SEC with PBS as the mobile phase on the Superdex 200 column or the Superose six Increase 10/300 GL for gH/gL-C4b and gH/gL-ferritin, respectively. gH/gL-IMX313 was flash frozen and stored at −80°C. gH/gL-ferritin was expressed and purified within a week of each immunization and stored at 4°C.

#### Purification of gH/gL-I3

To prepare an affinity chromatography resin to purify gH/gL-I3, 4.5 mg E1D1 antibody was incubated with 1 mL Protein A resin with rotation at room temperature for 30 min and then washed thoroughly with PBS. 6.5 mg of disuccinimidyl suberate was dissolved in 0.5 mL DMSO, then diluted in 10 mL PBS and added to the Protein A resin. The resin and DSS mixture were incubated at room temperature with rotation for at least 1 h. The resin was washed thoroughly with PBS, and then incubated overnight with rotation at 4°C in 10 mL of 1 M Tris, pH 7.5, and washed again extensively with PBS. The resin was then washed with Pierce IgG Elution buffer to remove any E1D1 antibody that was not crosslinked to the resin, and then washed again with PBS. The E1D1 affinity resin was stored in 50 mM Tris, 150 mM NaCl, 0.02% azide when not in use.

Supernatant from cells transduced with gH/gL-I3-was incubated with the E1D1 resin, washed with TBS, eluted with Pierce IgG elution buffer, and neutralized with a 1/10^th^ volume of 1 M Tris pH 8. The eluted protein was further purified by SEC using a Superose six Increase column with 50 mM Tris, 150 mM NaCl, 150 mM L-arginine, pH 8 as the mobile phase. Purified protein was flash frozen and stored at −80°C.

#### Size exclusion chromatography with multi-angle light scattering

Fractions containing single predominant species from the initial round of size exclusion chromatography were concentrated down with 10,000 MWCO protein concentrators (Novagen) to a concentration of 1.0–2.0 mg/mL. 100 uL of each sample was then run through a high-performance liquid chromatography system (Agilent 1260) using a Superdex 200 10/300 GL or a Superose six Increase 10/300 column gel filtration column at an elution rate of 1 mL/min in Pierce TBS in line with a multi-angle light scattering detector (Wyatt Heleos) and refractive index detector (Wyatt tREX). The data was then analyzed using ASTRA (Wyatt Technologies) to calculate the absolute molecular weights for each designed protein. Accounting for error in light scattering data acquisition, species with calculated molecular weights within 13% of the expected target molecular weight for each design were considered to be forming the anticipated oligomeric state.

#### Recombinant antibodies

Cloning, expression and purification of AMMO1,^55^ CL40, and CL59^59^ was performed as previously described. For cloning of E1D1, codon-optimized cDNA corresponding to E1D1 VH (GenBank: KX755644) was synthesized (Integrated DNA Technologies) and cloned in-frame with the human IgG1 constant region in pTT3-based expression vectors. Codon-optimized cDNA corresponding to E1D1 VL (GenBank: KX755645) was cloned in-frame with the human kappa constant regions in pTT3-based expression vectors. Recombinant E1D1 was expressed in 293-E cells and purified using Protein A affinity chromatography.

#### Negative-stain electron microscopy

For gH/gL 4-mer and 7-mer, 1% uranyl formate negative staining solution and Formvar/carbon grids (Electron Microscopy Sciences) of 300 mesh size were used to perform the negative staining experiment. The protein samples of 4-mer and 7-mer gH/gL were diluted to ∼40 μg/mL and ∼50 μg/mL, respectively and applied for 60 s on glow discharged grids. Excess sample was blotted off using Whatman filter paper and the grids were rinsed using water droplets and further stained for additional 60 s. Excess stain was blotted off and the grids were air dried for 1–2 min.

For gH/gL 24-mer and 60-mer, sample were diluted to 100 μg/mL and 3 μL was negatively stained using Gilder Grids overlaid with a thin layer of carbon and 2% uranyl formate as previously described.^93^

For the gH/gL 4-mer and 7-mer, data were collected using a FEI Tecnai T12 electron microscope operating at 120 keV equipped with a Gatan Ultrascan 4 × 4K CCD camera. The images were collected using an electron dose of 45.05 e−/Å^2^, a magnification of 67,000× that corresponds to pixel size of 1.6 Å, and exposure time of 1 s. The defocus range used was −1.00 μm to −2.00 μm. The data was collected using Leginon interface^94^ and processed using cryoSPARC.^95^ Particles were further picked from the micrographs and subjected to 2D classification and the best 2D classes were selected.

For the gH/gL 24-mer, data were collected on an FEI Technai 12 Spirit 120kV electron microscope equipped with a Gatan Ultrascan 4000 CCD camera. A total of 150 images were collected per sample by using a random defocus range of 1.1–2.0 μm with a total exposure of 45 e−/A.^2^ Data were automatically acquired using Leginon, and data processing was carried out using Appion.^96^ The parameters of the contrast transfer function (CTF) were estimated using CTFFIND4,^97^ and particles were picked in a reference-free manner using DoG picker.^98^ Particles were extracted with a binning factor of 2 after correcting for the effect of the CTF by flipping the phases of each micrograph with EMAN 1.9.^99^ The gH/gL 24-mer stack was pre-processed in RELION/2.1^100^, ^101^, ^102^ with an additional binning factor of two applied, resulting in a final pixel size of 6.4 Å. Resulting particles were sorted by reference-free 2D classification over 25 iterations.

For the 60-mer, data were collected on an Talos L120C 120kV electron microscope equipped with a CETA camera. A total of ∼350 images were collected per sample by using a random defocus range of 1.3–2.3 μm, with a total exposure of 35 e−/A^2^, and a pixel size of 3.16 Å/pixel. Data were automatically acquired using EPU (ThermoFisher Scientific). All data processing was performed using CryoSPARC.^95^ The parameters of the contrast transfer function (CTF) were estimated using CTFFIND4,^97^ and particles were picked initially in a reference-free manner using blob picker, followed by template picking using well-defined 2D classes of intact nanoparticles. Particles were extracted after correcting for the effect of the CTF for each micrograph with a box size of 256 pixels. Extracted particles were sorted by reference-free 2D classification over 20 iterations. 3D ab initio was performed in cryoSPARC with the subsequent homogeneous refinement step performed using icosahedral symmetry. The resulting 3D map was displayed at two different contours levels and images were generated using ChimeraX.^103^

#### Immunizations in C57BL/6 mice

Comparative immunogenicity studies were performed in groups of 10C57BL/6 mice (5 male and five female) between 7 and 10 weeks of age. After collecting a pre-bleed, mice were immunized at weeks 0, 4, and 12 with 5 μg (total protein) of monomer, 4-mer, 7-mer, 24-mer, or 60-mer formulated with 20% (v/v) synthetic lipid A in squalene emulsion SLA-SE^104^ in PBS or TBS (60-mer only) at a total volume of 100 μL. Mice were immunized via intramuscular injection split into two 50 μL doses split between both rear legs. Blood was collected retro-orbitally 2 weeks after the first and second immunizations and via cardiac puncture at week 14. Blood was collected in tubes containing a 1/10^th^ volume of citrate. Plasma was separated from whole blood via centrifugation and then heat inactivated at 56°C for 30 min. For passive transfer experiments into humanized mice immunizations were performed in groups of 20C57BL/6 mice (10 male and 10 female) between 7 and 10 weeks of age. After collecting a pre-bleed, mice were immunized at weeks 0 and 4 with 5 μg of gH/gL monomer or 60-mer formulated in PBS (monomer) or TBS (60-mer) with 50% (v/v) Sigma Adjuvant System (SAS) for a total volume of 100 μL. Mice were immunized via intramuscular injection split 50 μL each between both rear legs. Blood was collected retro-orbitally via cardiac puncture at week six into a separate vial for each mouse containing 100 μL citrate. Plasma was separated from whole blood via centrifugation.

#### IgG purification from murine plasma

Plasma was pooled and heat inactivated at 56°C for 1 h then diluted in protein G binding buffer and passed over a column containing 1mL of protein A/G resin. The column was then washed 3 times with five column volumes of binding buffer. Finally, IgG was eluted from the resin in 5 × 2 mL fractions using IgG elution buffer. Fractions were buffer exchanged into PBS, concentrated, filter sterilized, and yields were measured by nanodrop.

#### EBV-reporter virus production

To produce B-cell tropic GFP reporter viruses (B95-8/F), 9×10^6^,293–2089 cells were seeded on a 15 cm tissue culture plate in cRPMI containing 100 μg/mL hygromycin. 24 h later the cells were washed twice with PBS, the media was replaced with cRMPI without hygromycin, and the cells were transfected with 15 μg of each of p509 and p2670 expressing BZLF1 and BALF4, respectively, using GeneJuice transfection reagent.^91^^,^^105^ 72 h later the cell supernatant was collected, centrifuged at 300 × *g* for 5 min and then passed through a 0.8 μm filter. To produce epithelial cell tropic virus, B cells harboring AKATA-GFP EBV were suspended at 4×10^6^ cells/mL in RPMI containing 1% FBS. Goat anti-human IgG was added to a final concentration 100 μg/mL and incubated at 37°C for 4 h. Cells were then diluted to 2×10^6^ cells/mL in RPMI containing 1% FBS and incubated for 72 h. Cells were pelleted by centrifugation at 300 × *g* for 10 min and then the supernatant was passed through a 0.8 μm filter. Bacitracin was added to a final concentration of 100 μg/mL. Virions were concentrated 25× by centrifugation at 25,000 × *g* for 2 h and re-suspended in RPMI containing 100 μg/mL bacitracin. Virus was stored at −80°C and thawed immediately before use.

#### B cell neutralization assay

B cell neutralization assays were carried out in Raji cells essentially as described.^38^ Mouse plasma was serially diluted in duplicate wells of 96 well round-bottom plates containing 25 μL of cRPMI. 12.5 μL of B95-8/F virus (diluted to achieve an infection frequency of 1–5% at the final dilution) was added to each well and plates were incubated at 37°C for 1 h. 12.5 μL of cRPMI containing 4×10^6^ Raji cells/mL was added to each well and incubated for another hour at 37°C. The cells were then pelleted, washed once with cRPMI, and re-suspended in cRPMI. Reciprocal plasma dilutions are reported relative to the final infection volume (50 μL). After 3 days at 37°C, cells were fixed in 2% paraformaldehyde. The percentage of GFP + Raji cells was determined on a BD LSRII cytometer or Luminex Guava HT cytometer.

To account for any false positive cells due to auto-fluorescence in the GFP channel, the average %GFP + cells in negative control wells (n = 4–6) was subtracted from each well. The infectivity (%GFP+) for each well was plotted as a function of the plasma dilution. The neutralization curve was fit using the log(inhibitor) versus response-variable slope (four parameters) analysis in Prism 9.2.0. The half maximal inhibitory plasma dilution ID_50_ was interpolated from the curve in Prism 9.2.0.

For depletion assays, the average %GFP + cells in negative control wells (n = 4–6) was subtracted from each well. The %Infectivity was calculated for each well by dividing the %GFP + cells in each well by the average %GFP + cells in the most dilute plasma dilution wells and multiplying by 100. % Infectivity was plotted as a function of the plasma dilution. The neutralization curve was fit using the log(inhibitor) versus response-variable slope (four parameters) analysis in Prism 9.2.0.

#### Epithelial cell neutralization assay

1.5 × 10^4^ SVKCR2 cells per well were seeded into a 96 well tissue culture plate. The following day plasma was serially diluted in duplicate wells containing 20 μL of media in a 96 well flat bottom plate followed by the addition of 20 μL of 25× concentrated epithelial cell-tropic virus and incubated for 15 min. Media was aspirated from the SVKCR2 cells and replaced by the antibody-virus mixture and incubated at 37°C. 48 h later the cells were detached from the plate using 0.25% trypsin, transferred to a 96 well round bottom plate, washed twice with PBS, and fixed with 10% formalin, and the percentage of GFP + cells were determined on an BD LSRII cytometer or Luminex Guava HT. Percent neutralization was determined as in the B cell neutralization assay.

#### Measurement of plasma antibody endpoint binding titers by anti-His capture ELISA

30μL/well of rabbit anti-His tag antibody was adsorbed at a concentration of 0.5 μg/mL on to 384 well microplates at 4°C for 16 h in a solution of 0.1 M NaHCO3 pH 9.4–9.6 (coating buffer). The next day, plates were washed 4 times with one x PBS, 0.02% Tween 20 (ELISA wash buffer) prior to blocking for 1 h with 80 μL/well of 1× PBS containing 10% non-fat milk and 0.02% Tween 20 (blocking buffer). After blocking, plates were washed 4× with wash buffer and 30 μL/well of a 2 μg/mL solution of monomeric His-tagged gH/gL diluted in blocking buffer was added to the plate and incubated for 1 h, and then washed 4× with ELISA wash buffer. Plasma was diluted in blocking buffer and 3-fold serial dilutions were performed in duplicate followed by a 1-h incubation at 37°C. 8–16 additional control wells were included that contained immobilized gH/gL but no immune plasma (control wells). Following four additional washes with ELISA wash buffer, a 1:2,000 dilution of goat anti-mouse IgG-HRP (SouthernBiotech) in blocking buffer was added to each well and incubated at 37°C for 1 h followed by four washes with ELISA wash buffer. 30 μL/well of SureBlue Reserve TMB Microwell Peroxidase substrate was added. After 5 min, 30μL/well of 1N sulfuric acid was added and the A_450_ of each well was read on a Molecular Devices SpectraMax M2 plate reader. The binding threshold was defined as the average plus 10 times the SD of the determined by calculating the average of A_450_ values of the control wells. Endpoint titers were interpolated from the point of the curve that intercepted the binding threshold using the Prism 9.2.0 package.

#### Measure of competitive binding titers by ELISA

Coating, blocking, and gH/gL immobilization steps were performed as described under “Measurement of plasma antibody endpoint binding titers by anti-His capture ELISA.” Following capture of monomeric gH/gL, equal amounts of plasma from each mouse in a group were pooled and diluted in blocking buffer and 2-fold serial dilutions were performed, followed by a 1-h incubation at 37°C. Following four additional washes with ELISA wash buffer, monoclonal antibodies AMMO1, CL40, CL59, and E1D1 were added at a concentration that achieves half-maximal binding (EC_50_; pre-determined in the same assay in the absence of competing sera) to each well containing serially diluted pooled sera from each group, followed by a 1-h incubation at 37°C. After four washes with ELISA washing buffer, a 1:20,000 dilution of goat anti-human IgG-HRP (Jackson ImmunoResearch) in blocking buffer was added to each well and incubated at 37°C for 1 h followed by four washes with ELISA wash buffer. Addition of SureBlue Reserve TMB Microwell Peroxidase substrate, addition of 1N sulfuric acid, and reading of plates was performed as described above. The average A_280_ values of buffer only control wells were subtracted from each mAb containing well and plotted in Prism 9.2.0. A_280_ values were plotted as a function of the log_10_ of the plasma dilution. A binding curve was fit using the Sigmoidal, 4PL, X is log(concentration) least squares fit function. Maximum binding was defined as the best-fit value for the top of each curve computed in Prism. A_280_ values at each dilution on the curve were divided by the maximum binding and multiplied by 100 to calculate the % of max binding ([A_280_ at each dilution/max binding] × 100). The titer at which half-maximal binding was observed was interpolated from the binding curve using the Prism 9.2.0 package (GraphPad Software).

#### Biotinylation of recombinant proteins

Recombinant gH/gL proteins were biotinylated using the EZ-Link NHS-PEG4-Biotin Kit according to the manufacturer’s instructions. The biotinylation reaction incubated overnight at 4°C, after which excess biotin was removed using a Zeba Spin Desalting Column.

#### Neutravidin capture ELISA

30 μL/well of a 0.3 μg/mL solution of NeutrAvidin in ELISA coating buffer was incubated on 384 well microplates at 4°C for 16 h. The next day, plates were washed 4 times with ELISA wash buffer prior to blocking for 1 h with 80 μL/well of 1X PBS containing 3% BSA and 0.02% Tween 20 (neutravidin blocking buffer). After blocking, plates were washed 4 times with ELISA wash buffer and 30 μL/well of a 2 μg/mL solution of biotinylated gH/gL monomer, 4-mer, 7-mer, 24-mer, or 60-mer was added and allowed to incubate 1 h. After four washes with ELISA wash buffer, a panel of monoclonal antibodies were diluted to 10 μg/mL in neutravidin blocking buffer and 3-fold serial dilutions were performed in duplicate followed by a 1-h incubation at 37°C. 8–16 additional control wells were included that contained immobilized the gH/gL but no monoclonal antibodies (control wells). Following four additional washes with ELISA wash buffer, a 1:5000 dilution of goat anti-human IgG-HRP (SouthernBiotech) in neutravidin blocking buffer was added to each well and incubated at 37°C for 1 h followed by four washes with ELISA wash buffer. Addition of SureBlue Reserve TMB Microwell Peroxidase substrate, addition of 1N sulfuric acid, and reading of plates was performed as described above.

#### Measurement of total plasma IgG

Plasma was serially diluted in ELISA coating buffer in duplicate and incubated on 384-well microplates at 4°C for 16 h. At least 10 additional control wells were included that contained only coating buffer and no plasma. The next day, plates were washed 4× with ELISA wash buffer prior to blocking for 1 h with 80 μL/well of ELISA blocking buffer. After blocking, plates were washed 4× with ELISA wash buffer and a 1:4000 dilution of goat anti-mouse IgG Human ads-HRP in ELISA blocking buffer was added to each well and incubated at 37°C for 1 h followed by four washes with ELISA wash buffer. Addition of SureBlue Reserve TMB Microwell Peroxidase substrate, addition of 1N sulfuric acid, and reading of plates was performed as described above.

#### Bead depletion assays

To conjugate biotinylated gH/gL and gH/gL-KO to beads, streptavidin magnetic beads were washed 2× with PBS using a magnetic separator and then co-incubated with biotinylated gp350, gH/gL, or gH/gL-KO on a rotator overnight at 4°C. The supernatant was collected using a magnetic separator and analyzed via spectrophotometry to ensure protein concentration in supernatant had been reduced and saturation of beads was achieved. Beads were washed 2× to remove excess unbound gp350, gH/gL, or gH/gL-KO and stored at 4°C in PBS.

For depletion of plasma antibodies, beads were re-suspended with diluted, pooled plasma and incubated 16 h at 4°C on a rotator. Beads were then separated from plasma using a magnetic separator and the remaining plasma was collected and transferred to a new tube and subsequently tested for binding to gH/gL and for neutralizing activity.

#### EBV challenge in humanized mice

10 weeks post-cell transfer of CD34-enriched PBSCs, successful human cell engraftment in NSG mice was confirmed via immunophenotyping of circulating lymphocytes using antibodies at indicated dilution: hCD45-FITC (1:100), hCD8-BV21 (1:100), L/D-BV506 (1:200), hCD19-BV711 (1:100), hCD20-BV786 (1:200), mCD45-APC (1:200), hCD4-AF700 (1:250), hCD33-PE (1:100), mCD16/32 (1:200).

12–13 weeks post-human HSPC transfer, 500 μg of total IgG purified from immunized C57BL/6 mice were injected per humanized mouse intraperitoneally (IP). Two days prior to, and one day following transfer, blood was collected to measure the relative levels of total and anti-gH/gL IgG in the plasma.

48 h after transfer, the mice received a dose of EBV B95.8/F, equivalent to 33,000 infectious units as determined by infection of Raji cells, via retro-orbital injection. Beginning 3 weeks post-challenge (Day 21), peripheral blood samples were collected weekly to determine the presence of EBV DNA in whole blood and to immunophenotype circulating lymphocytes.Mice were weighed three times a week on non-consecutive days. If mice fell below 80% of their starting weight, or met other criteria for symptoms of pain (i.e. hunching, lack of mobility, etc.), they were euthanized.

Levels of EBV in the blood were monitored on a weekly basis using primers specific for BALF5 as described in “Quantitative PCR analysis of human cells in huCD34 engrafted mice.” Blood samples were collected from mice on day prior to challenges and weekly beginning 3 weeks post-challenge (day 21) through to the end of the experiment at 10 weeks post-challenge (day 70), or until the animals reached euthanasia criteria. Spleens were harvested from each mouse at the day 70 endpoint, or earlier if they met euthanasia criteria.

Ten weeks post-challenge, surviving mice were euthanized and spleens were collected and weighed. DNA was extracted from 5×10^6^ total splenocytes, utilizing the DNeasy Blood & Tissue Kit and according to the manufacturer’s instructions, for subsequent viral load analysis.

#### Quantitative PCR analysis of human cells in HuCD34 engrafted mice

A primer-probe mix specific for the EBV BALF5^106^ gene was used to quantify EBV in DNA extracted from blood or spleen in hCD34 engrafted NSG recipient mice at the time points described. Each 25 μL qPCR reaction contained 12.5 μL of 2× QuantiTect Probe PCR Master Mix, 600nM of each primer, 300nM of FAM-labeled probe, 1.25 μL of a TaqMan 20× VIC-labeled RNase-P primer-probe mix. Reactions were heated to 95 °C for 15 min to activate DNA polymerase followed by 50 cycles of 95°C for 15 s 60°C for 60 s, on an Applied Biosystems QuantStudio seven Flex Real-Time PCR System. Synthetic DNA fragments containing the BALF5 target gene as well as flanking genomic regions were synthesized as double stranded DNA gBlocks, and were used to generate a standard curve with known gene copy numbers ranging from 10^2^–10^7^ copies/mL. The copy number in extracted DNA was determined by interpolating from the standard curve. Serial dilutions of reference standard were used to experimentally determine a limit of detection of 6.25 copies, which corresponds to the amount of template that can be detected in >95% of reactions. For graphical purposes, samples with no amplification or those yielding values below the limit of detection were assigned a value of 0.625 copies.

#### Biolayer interferometry

BLI assays were performed on the Octet Red 96 instrument at 30°C with shaking at 1,000 RPM. Anti-Human Fc Capture (AHC) Biosensorswere submerged in wells of black 96-well microplates (containing 250 μL of kinetics buffer (PBS, 0.02%Tween 20, 0.03% azide, 0.1% BSA) for at least 15 min prior to any data collection. Biosensors were submerged for 30 s in KB to establish baseline response (baseline step 1). Biosensors were submerged in KB containing 10 μg/mL of monoclonal antibodies for 240 s (load step). Biosensors were then equilibrated for 60 s in kinetics buffer alone (baseline step 2), after which the antibody-bound biosensors were submerged in wells containing a 250 nM solution of gH/gL or gH/gL-KO in KB for 300 s (association step) followed by immersion in KB for 300 s (dissociation step).

The background signal from each analyte-containing well was measured using empty reference sensors and subtracted from the signal obtained with each corresponding ligand-coupled sensor at every timepoint.

### Quantification and statistical analysis

Kruskal-Wallis tests were performed to assess whether the distributions of responses varied across treatment groups, with p values < 0.05 considered significant. If the Kruskal-Wallis test reached significance, a Mann-Whitney test was used to compare the distribution of outcomes between the pairs of groups considered. Immunogenicity was compared across each pair of treatment groups; for spleen weights and viral DNA copies, each group was compared to the infected control. The Holm method was used to adjust for multiplicity across the Mann-Whitney tests conducted for each outcome, with Holm’s adjusted p values reported. For survival data, significant differences were determined using Log rank Mantel-Cox test.

## Data Availability

• Data: The published article includes all datasets generated or analyzed during this study. • Code: The published article does not report custom computer code. • Any additional information required to reanalyze the data reported in this paper is available from the lead contact upon request.

## Supplemental information

Document S1. Figures S1–S5 and Tables S1 and S2

Document S2. Article plus supplemental information
