# Supplementary material for: Immunization with a self-assembling nanoparticle vaccine displaying EBV gH/gL protects humanized mice against lethal viral challenge
Source: Cell Rep Med. 2022 Jun 14;3(6):100658. doi: 10.1016/j.xcrm.2022.100658 (PMC9245003; doi:10.1016/j.xcrm.2022.100658)
Supplement: Document S1. Figures S1–S5 and Tables S1 and S2 [file mmc1.pdf]

Cell Reports Medicine, Volume *3*

Supplemental information

Immunization with a self-assembling nanoparticle vaccine displaying EBV gH/gL protects humanized mice against lethal viral challenge

Harman Malhi, Leah J. Homad, Yu-Hsin Wan, Bibhav Poudel, Brooke Fiala, Andrew J. Borst, Jing Yang Wang, Carl Walkey, Jason Price, Abigail Wall, Suruchi Singh, Zoe Moodie, Lauren Carter, Simran Handa, Colin E. Correnti, Barry L. Stoddard, David Veesler, Marie Pancera, James Olson, Neil P. King, and Andrew T. McGuire

Cell Reports Medicine, Volume *3*

Supplemental information

Immunization with a self-assembling nanoparticle vaccine displaying EBV gH/gL protects humanized mice against lethal viral challenge

Harman Malhi, Leah J. Homad, Yu-Hsin Wan, Bibhav Poudel, Brooke Fiala, Andrew J. Borst, Jing Yang Wang, Carl Walkey, Jason Price, Abigail Wall, Suruchi Singh, Zoe Moodie, Lauren Carter, Simran Handa, Colin E. Correnti, Barry L. Stoddard, David Veesler, Marie Pancera, James Olson, Neil P. King, and Andrew T. McGuire

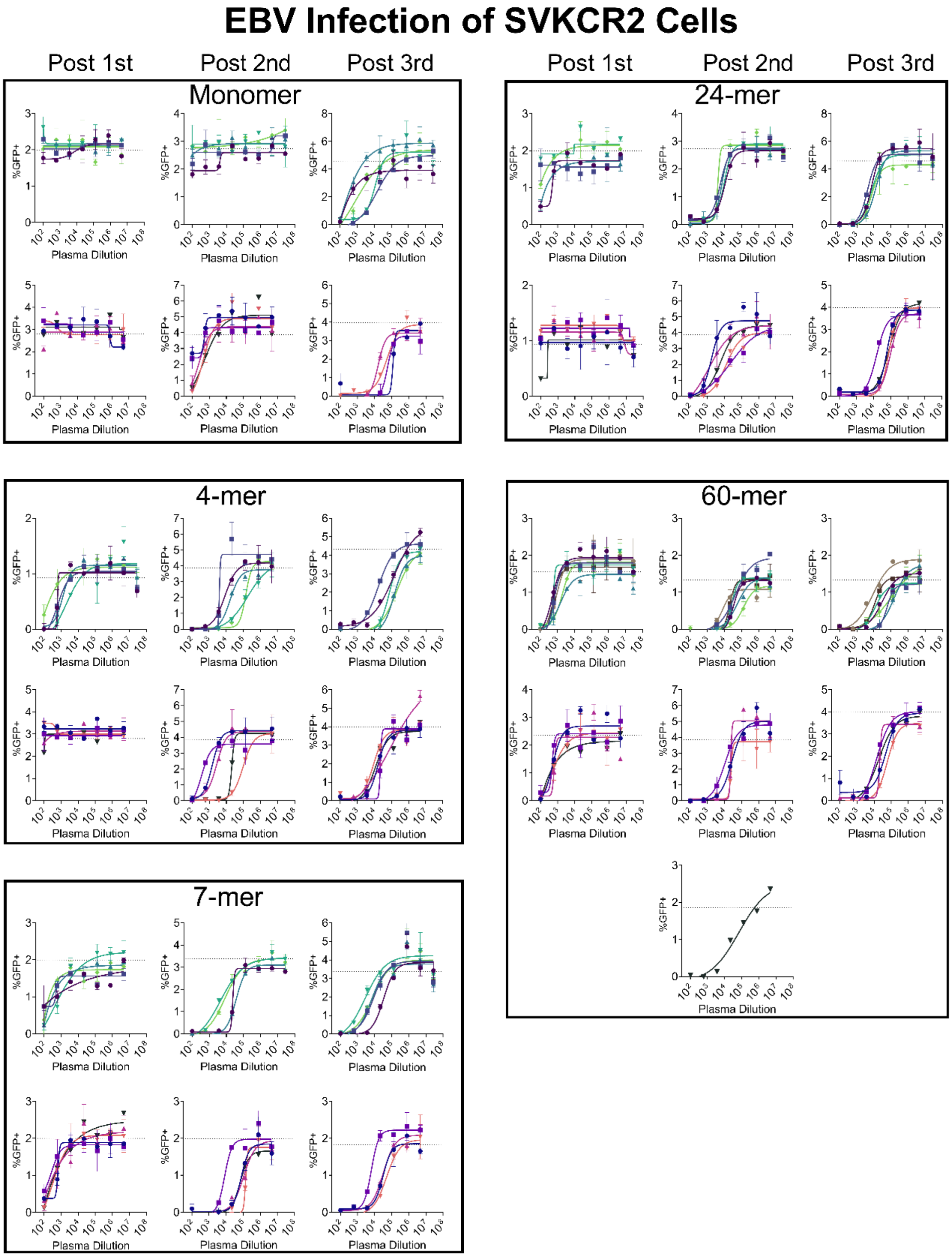

Figure S1 Related to Figure 2. Inhibition of EBV infection of epithelial cells by gH/gL immune plasma. Plasma from C57BL/6 (n=10-12 per group) mice were serially diluted and evaluated for their ability to inhibit AKATA-GFP EBV infection of SVKCR2 cells. Animals immunized with the same monomeric gH/gL or gH/gL nanoparticles are bound by boxes. The y-axis shows the % of background subtracted GFP+ SVKCR2 cells and the y-axis is the plasma dilution. Each data point represents mean and error bars represent the standard deviation of two technical replicates. Different colored symbols represent the same individual mouse in each group at the indicated timepoints The dashed line indicates the % of GFP+ SVKCR2 cells in the absence of plasma.

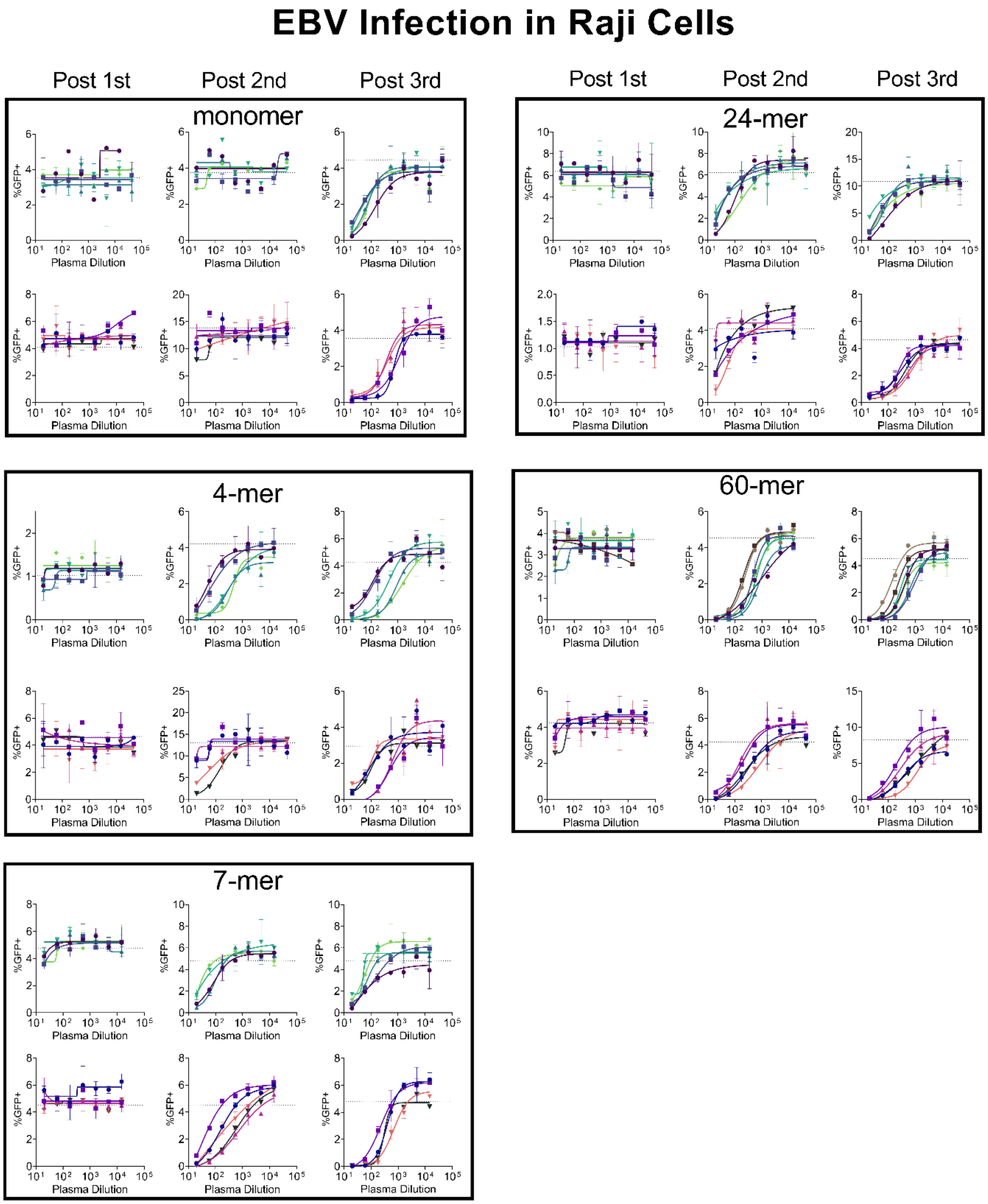

Figure S2. Related to Figure 2. Inhibition of EBV infection in B cells by gH/gL immune plasma. Plasma from C57BL/6 mice (n=10-12 per group) were serially diluted and evaluated for their ability to inhibit EBV B95.8/F infection of Raji cells. Animals immunized with the same monomeric gH/gL or gH/gL nanoparticles are bound by boxes. The y-axis shows the % of background subtracted GFP+ Raji cells and the y-axis is the plasma dilution. Each data point represents mean and the error bars represent the standard deviation of two technical replicates. Different colored symbols represent the same individual moue in each group at the indicated timepoints. The dashed line indicates the % of GFP+ Raji cells in the absence of plasma.

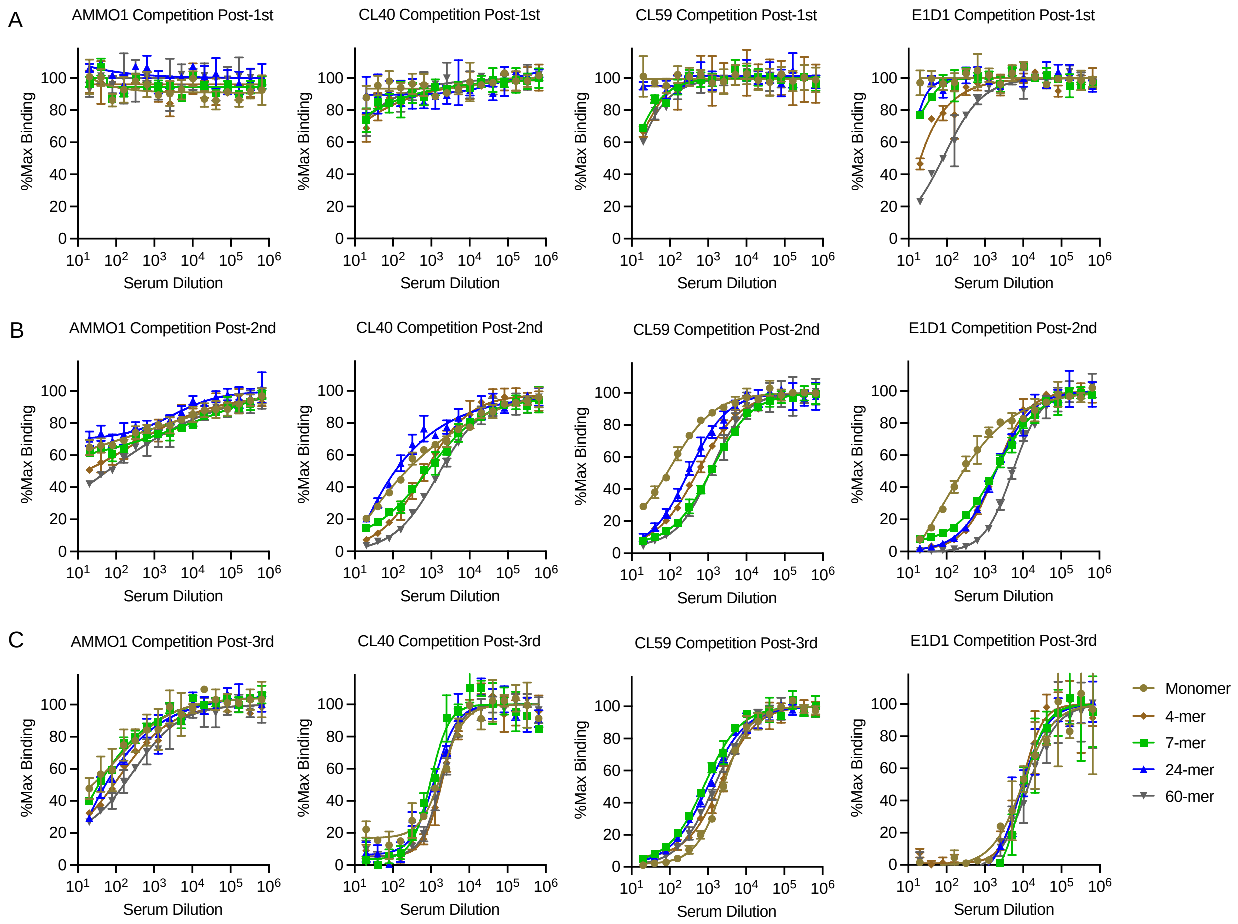

Figure S3. Related to Figure 3. Competitive binding between immune plasma and monoclonal antibodies. Competitive binding ELISAs were performed using pools of plasma from groups of C57BL/6 mice (n=10-12 per group) immunized with monomeric gH/gL or multimeric gH/gL nanoparticles, and a panel of anti-gH/gL antibodies. At each time point, pooled sera from each group were titrated on to gH/gL immobilized on an ELISA plate, after which either AMMO1, CL40, CL59, or E1D1 antibodies were added at a concentration previously determined to achieve half maximal binding. Competitions were performed using plasma pools collected at Post-1st (**A**), Post-2nd (**B**), and Post-3rd timepoints (**C**). Each data point represents mean and error bars represent the standard deviation of two technical replicates.

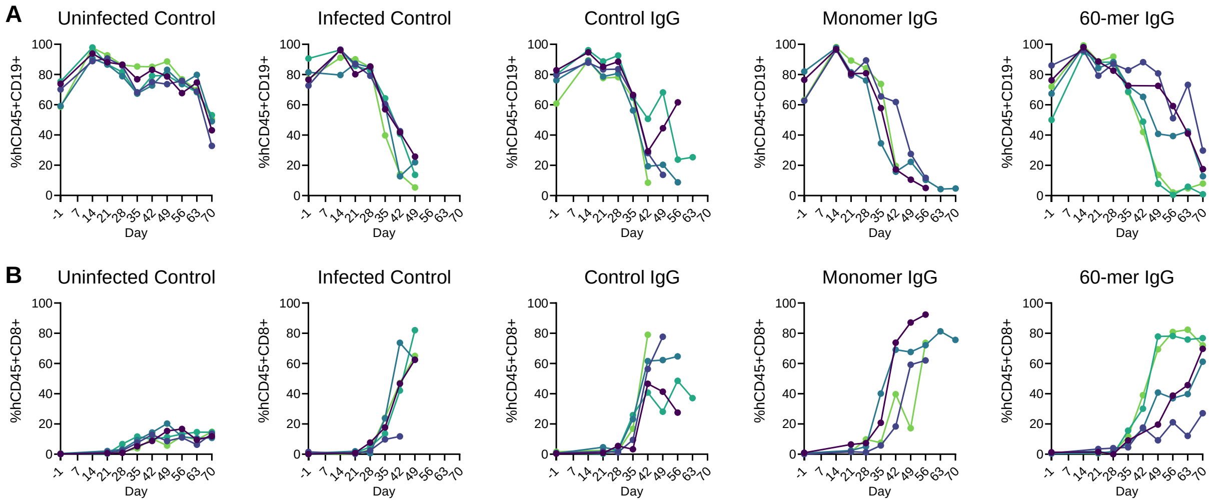

Figure S4. Related to Figure 5. hCD19+ and hCD8+cell frequencies in humanized mice challenged with EBV hCD45+CD19+ B cells (**A**) and hCD45+CD8+ T cell (**B**) frequencies were measured in the peripheral blood drawn from the mice in Figure 5 at the indicated timepoints via flow cytometry. Individual mice in each group are indicated by different colored symbols. The same color is used to indicate the hCD45+CD19+ and hCD45+CD8+ T cells from the same mouse each group in **A** and **B.** The days indicated on the x-axis are relative to the time of challenge (day 0).

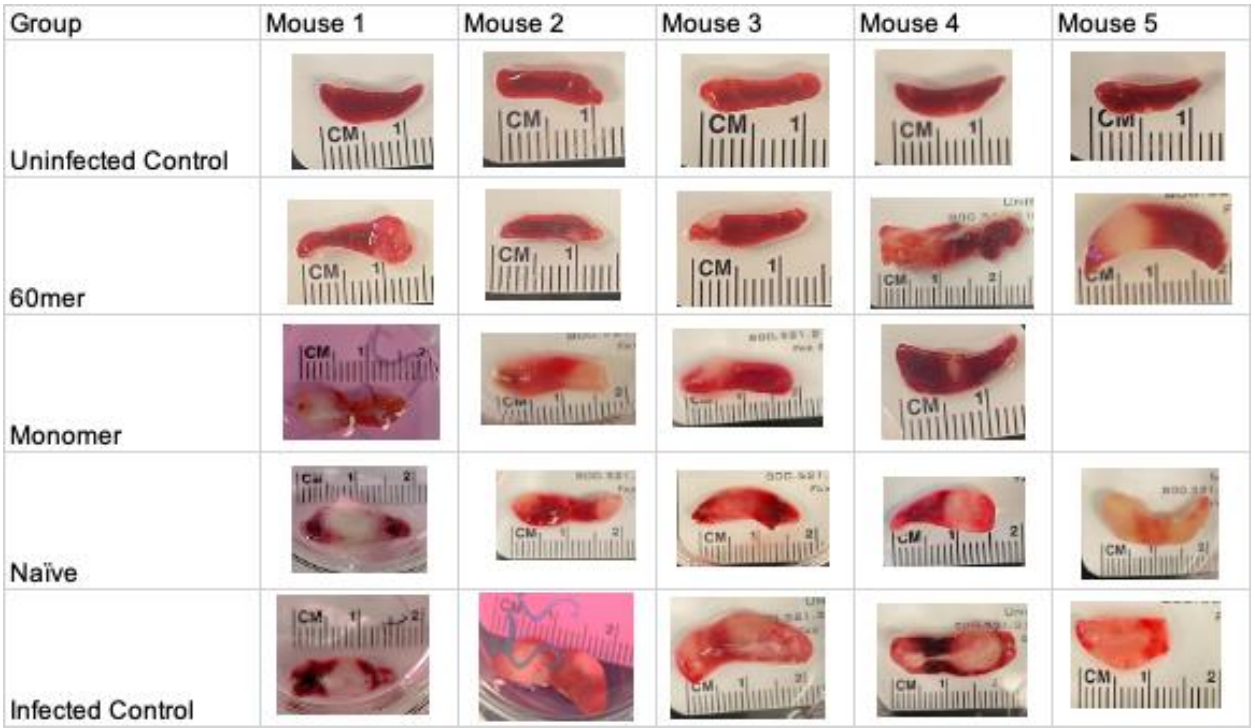

Figure S5. Related to Figure 5. Photographs of spleens from humanized mice challenged with EBV were taken at the time of necropsy.

Table S1. Yields of various gH/gL nanoparticles. Related to Figure 1.

gH/gL protein	Yield mg/L (mean ± S.D.)	# of production runs
Monomer	8.1	1
4-mer	2.6 ± 0.6	2
7-mer	1.8 ± 0.2	2
24-mer	1.4 ± 0.2	2
60-mer	0.5 ± 0.4	7

Table S2. Multimerization domains and observed and expected nanoparticle sizes. Related to Figure 1.

Multimerization domain	Sequence	Predicted MW of protomer (particle) kDa*	Observed MW of particle by SEC-MALS**
4-mer	GGGGSGGGGSC-(EAIKAAAELGKAGISSEEILELLRAAHELGLDP)×4 ECIKAAAELGKAGISSEEILELLRAAHELGLGGSHHHHHH	115.7 (462.8)	543 ± 76 kDa (S200)
IMX313, 7-mer	GSSKKQGDADVCGEVAYIQSVVSDCHVPTAELRTLLEIRKLFLEIQKLKVELQGLSKE	101 (700.7)	700 ± 89 kDa (S200)<br>635 ± 69 kDa (Superose 6)
H. pylori Ferritin, 24-mer	GGGGSGGGGSGGSGESQVRQQFSKDIEKLLNEQVNKEMQSSNLYMSMSSWCYTHSLDGAGLFLFDHAAEEYEHAKKLIIFLNENNVPVQLTSISAPEHKFEGLTQIFQKAYEHEQHISESINNIVDHAIKSKDHATFNFLQWYVAEQHEEEVLFKDILDKIELIGNENHGLYLADQYVKGIAKSRKSGS	114.9 (2757.6)	4418 ± 471 kDa (Superose 6)
Sec Opt I3, 60-mer	GSGSIEELFKKHKIVAVLRANSVEEAKKKALAVFLGGVDLIEITFTVPDADTVIKELSFLKEMGAIIGAGTVTSVEQARKAVESGAEFIVSPHLDEEISQFAKEKGVFYMPGVMTPTELVKAMKLGHTILKLFPGEVVGPQFVKAMKGPFPNVKFVPTGGVNLDNVAEWFKAGVQAVGVGEALNKGTPVEVAEKAKAFVEKIRGAT	155.7 (6942)	7377 ± 943kDa (Superose 6)

*Includes the weight of the peptide component predicted by https://web.expasy.org/protparam/ plus the weight of 8 putative N-linked glycosylation sites on gH/gL, each assigned a molecular weight of 1kDa

** the column used for size exclusion chromatography appears in parentheses.
